# Multiplex Networks of Cortical and Hippocampal Neurons Revealed at Different Timescales

**DOI:** 10.1371/journal.pone.0115764

**Published:** 2014-12-23

**Authors:** Nicholas Timme, Shinya Ito, Maxym Myroshnychenko, Fang-Chin Yeh, Emma Hiolski, Pawel Hottowy, John M. Beggs

**Affiliations:** 1 Department of Physics, Indiana University, Bloomington, Indiana, 47405, United States of America; 2 Santa Cruz Institute for Particle Physics, University of California Santa Cruz, Santa Cruz, California, 95064, United States of America; 3 Program in Neuroscience, Indiana University, Bloomington, Indiana, 47405, United States of America; 4 Department of Microbiology & Environmental Toxicology, University of California Santa Cruz, Santa Cruz, California, 95064, United States of America; 5 Physics and Applied Computer Science, AGH University of Science and Technology, 30–059, Krakow, Poland; Center of nonlinear, China

## Abstract

Recent studies have emphasized the importance of multiplex networks – interdependent networks with shared nodes and different types of connections – in systems primarily outside of neuroscience. Though the multiplex properties of networks are frequently not considered, most networks are actually multiplex networks and the multiplex specific features of networks can greatly affect network behavior (e.g. fault tolerance). Thus, the study of networks of neurons could potentially be greatly enhanced using a multiplex perspective. Given the wide range of temporally dependent rhythms and phenomena present in neural systems, we chose to examine multiplex networks of individual neurons with time scale dependent connections. To study these networks, we used transfer entropy – an information theoretic quantity that can be used to measure linear and nonlinear interactions – to systematically measure the connectivity between individual neurons at different time scales in cortical and hippocampal slice cultures. We recorded the spiking activity of almost 12,000 neurons across 60 tissue samples using a 512-electrode array with 60 micrometer inter-electrode spacing and 50 microsecond temporal resolution. To the best of our knowledge, this preparation and recording method represents a superior combination of number of recorded neurons and temporal and spatial recording resolutions to any currently available *in vivo* system. We found that highly connected neurons (“hubs”) were localized to certain time scales, which, we hypothesize, increases the fault tolerance of the network. Conversely, a large proportion of non-hub neurons were not localized to certain time scales. In addition, we found that long and short time scale connectivity was uncorrelated. Finally, we found that long time scale networks were significantly less modular and more disassortative than short time scale networks in both tissue types. As far as we are aware, this analysis represents the first systematic study of temporally dependent multiplex networks among individual neurons.

## Introduction

Understanding how large groups of neurons process and represent information in neural systems is a fundamental problem of neuroscience. One popular avenue to investigate the behaviors of large populations of neural sources is to analyze their connectivity [1]–[3]. Traditionally, these analyses have focused on individual networks that contain only one type of connection. However, recent work has shown the importance of interdependent networks [4]–[15]. These “multiplex networks” consist of multiple interdependent networks that share common nodes and possess different types of connections. In applications outside neuroscience, these previous studies frequently focused on the resilience properties of multiplex networks and on the properties of random multiplex networks.

In neuroscience applications, though rarely studied explicitly (see [13] as an exception), the multiplex properties of networks have often been examined in the context of comparing different types of connectivity. Neural connectivity has traditionally been conceptualized in three ways [16], [17]:

- Physical (or Structural or Anatomical) Connectivity: synapses, gap junctions, fiber bundles, etc.
- Functional Connectivity: statistical dependencies between the activities (action potentials, local field potentials, hemodynamic response, etc.) of the neural sources
- Effective (or Causal) Connectivity: time directed statistical dependencies of one neural source’s effect on the behavior of another neural source

All three types of connectivity have been widely studied in the literature (see [1]–[3], [18]–[23] for reviews). These types of connectivity form multiplex networks because they represent different connection types linking shared nodes. We are aware of only one study that explicitly researched multiplex networks of this type in neural systems [13], and that study was conducted on the level of brain region connectivity. Other studies have implicitly examined these multiplex networks on the level of brain region connectivity [22]–[27] and at the cellular level [28]–[31]. Typically, these studies have focused on the ability of one type of connectivity to predict another and what features, if any, of one type of connectivity are not represented in another type of connectivity.

While the investigation of multiplex networks in terms of physical, functional, and effective connectivity is certainly of great interest, we felt it would be productive to examine multiplex networks in the brain from a different point of view. The brain exhibits a large repertoire of neural phenomena over a wide range of time scales (e.g. EEG rhythms, action potentials, local field potentials, hemodynamic response, etc.). It has been argued that isolating phenomena at specific time scales (e.g. oscillations at different frequencies) and understanding their interactions are important to understanding how the brain integrates information [32]–[39]. Based on the existence of these phenomena, *we chose to examine multiplex networks of individual neurons with time scale dependent connections*. The neural phenomena listed above are constituted within or associated with the electrical activities of neural sources, and the time scales associated with physical connectivity between neurons are significantly shorter (less than ∼20 ms) than the time scales associated with many of these neural phenomena. Thus, to measure the interactions between neurons at the time scales associated with these neural phenomena, we chose to measure the effective connectivity between neurons at different time scales.

Previous studies have discussed the temporal multiplex properties of signals in the brain (e.g. multiple coding modalities) (see [34], [36], [37] for reviews). In terms of network connectivity, other studies have implicitly examined multiplex networks with time scale dependent functional connections ([40]–[44] for example) and effective connections ([27], [45] for example) at the level of brain regions. However, we are aware of only two other studies – both of which were conducted *in vitro* – that examined networks with time scale dependent connectivity at the cellular level [46]–[48]. Though these works implicitly examined multiplex networks, both studies treated networks at different time scales as distinct with essentially independent nodes and only one type of connection. In other words, these studies did not examine the uniquely multiplex properties of the networks under study, as we have done in this analysis.

In this work, we chose to use Transfer Entropy (TE) [49] to measure the time scale dependent effective connections between neurons. TE has been widely used in neural systems [45], [47], [50]–[62] and neural models [27], [63], [64]. TE measures how the state of one unit was changed or affected by the state of another unit. In our analysis, we used TE to determine the effective connectivity between individual neurons over isolated time scales ranging from sub-millisecond to seconds. Next, using graph theoretic methods [3], [41], [57], [58], [65]–[72], we analyzed the topology of the resulting networks. “Network topology” generally refers to the way in which the nodes (neurons in our case) in a network are connected. We used various network topology analysis tools to study the time scale dependent networks. In order to obtain the highest resolution electrophysiological recordings of as many individual neurons as possible, we used a state-of-the-art 512-electrode array to simultaneously record the spontaneous spiking activity of hundreds of neurons in organotypic cultures. *To the best of our knowledge, this preparation and recording method represents a superior combination of number of recorded neurons and temporal and spatial recording resolutions to any currently available in vivo system*.

We found several interesting results related to the topology of the measured effective connectivity networks. *First, we found that long time scale connections are independent of short time scale connections.* In other words, we found that the existence of a short time scale connection between two neurons was uncorrelated with the existence of a long time scale connection between those neurons. *Second, we found that highly connected neurons (so called “hubs”) tended to be isolated to specific time scales. Conversely, we found that non-highly connected neurons (“non-hubs”) did not tend to be isolated to a specific time scale.* These results represent the first evaluation of truly multiplex network properties at the neuron level and the hub result may have significant implications for fault tolerance in neural networks [4], [11]. *Third, we found network topology to be time scale dependent.* For instance, the physical distance between connected nodes was shown to increase as the time scale lengthened and the networks were shown to become more disassortative and less modular as the time scale lengthened. *Fourth, in some respects and at some time scales, hippocampal and cortical networks were shown to be significantly different, while in other respects and at different time scales they were very similar.* For instance, cortical networks where shown to be significantly more assortative than hippocampal networks for short time scales, but the two tissue types were similarly disassortative for longer time scales. ***These results represent the first systematic examination of time scale dependent multiplex networks of individual neurons and they indicate that these time scale dependent networks potentially differ by function and brain region.***

The results in this paper were presented in earlier versions at two conferences [73], [74].

## Results

### Electrophysiological Properties

**Figure 1:**
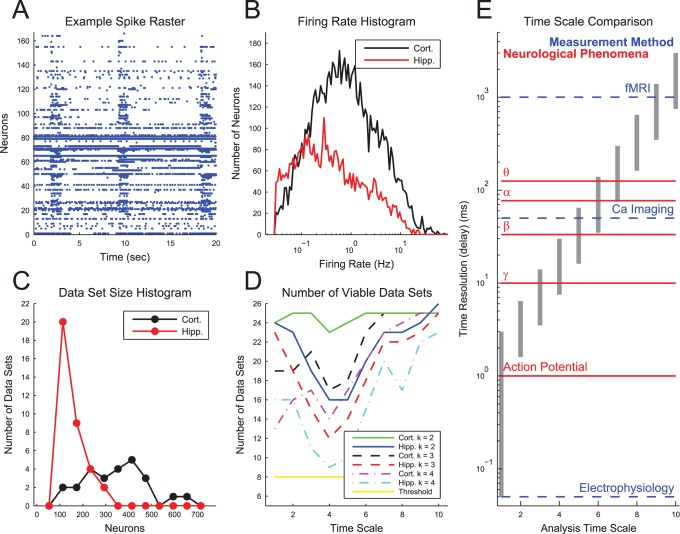
Analysis time scales and basic data properties. (**A**) Example spike raster from a cortical recording. (**B**) Firing rate histogram of neurons in all hippocampal and cortical recordings. (**C**) Histogram of the number of neurons in each recording. (**D**) The number of viable data sets or recordings. Data sets were deemed viable if they produced sub-networks with at least 50 neurons and a given average degree (k). We used sub-networks with k = 3 throughout the analysis. (**E**) Time resolutions for the 10 discrete time scales used in this analysis in comparison to the approximate time scales for various neurological phenomena and measurement methods. Note that some measurement methods (e.g. MEG and EEG (forms of electrophysiology), as well as fMRI) are not capable of recording the activity of individual neurons, unlike calcium imaging or cellular electrophysiology (as was used in this study). The analysis time scales were chosen to logarithmically span many neurological time scales and they allowed us to compare network structure on this wide range of time scales. Note that the analysis time scales overlap to ensure that all phenomena are adequately measured.

We performed 25 cortical and 35 hippocampal recordings from mouse cortico-hippocampal organotypic cultures using a 512-electrode array with a sampling rate of 20 kHz. These recordings were pre-processed and spike sorted to yield spike times for each neuron (see *Materials and Methods*). The spiking activity of the neurons was dominated by bursts of activity (Fig. 1 A and S1 Fig). The neurons in the cortical recordings had a mean firing rate of 2.10 Hz, while the neurons in the hippocampal recordings had a mean firing rate of 1.21 Hz (Fig. 1 B). On average, we found 309.4 spike sorted neurons in each cortical recording (7735 total neurons) and 120.4 spike sorted neurons in each hippocampal recording (4214 total neurons) (Fig. 1 C). The cortical recordings yielded significantly more neurons, perhaps because the hippocampal tissues were physically smaller in size.

### Transfer Entropy Analysis and Connectivity Properties

After obtaining the spike times for the neurons, we used transfer entropy (TE) to measure the effective connectivity between individual neurons over 10 isolated time scales ranging from sub-millisecond to seconds (Fig. 1 E, see *Materials and Methods*). Since this study was relatively exploratory, we chose these 10 time scales to logarithmically span many time scales relevant for neural phenomena. For instance, the analysis was able to capture interactions on the time scale visible to fMRI (∼1 second), as well as interactions on the time scale of individual action potentials (∼1 millisecond). This allowed us to compare the network structure on the fMRI time scale to the network structure on the individual action potential time scale. We would like to emphasize that other time scales could be chosen for other applications.

To evaluate which connections were significant, we compared the TE value found in the data to the TE values obtained from 5000 jittered spike time series. The jittering procedure consisted of randomly altering the time of each spike by a small amount proportional to the time scale being investigated. Thus, the jittering procedure preserved the overall firing rate of each neuron and the long time scale changes in neuron firing rates (i.e. bursts), but removed the precise spike timing between neurons. The percentage of the TE values from jittered data that were larger than the TE value obtained from the data was taken as the p-value. Only connections with p<0.001 were used in the analysis. We then treated the significant connections as binary edges in the networks and the neurons as nodes.

**Figure 2:**
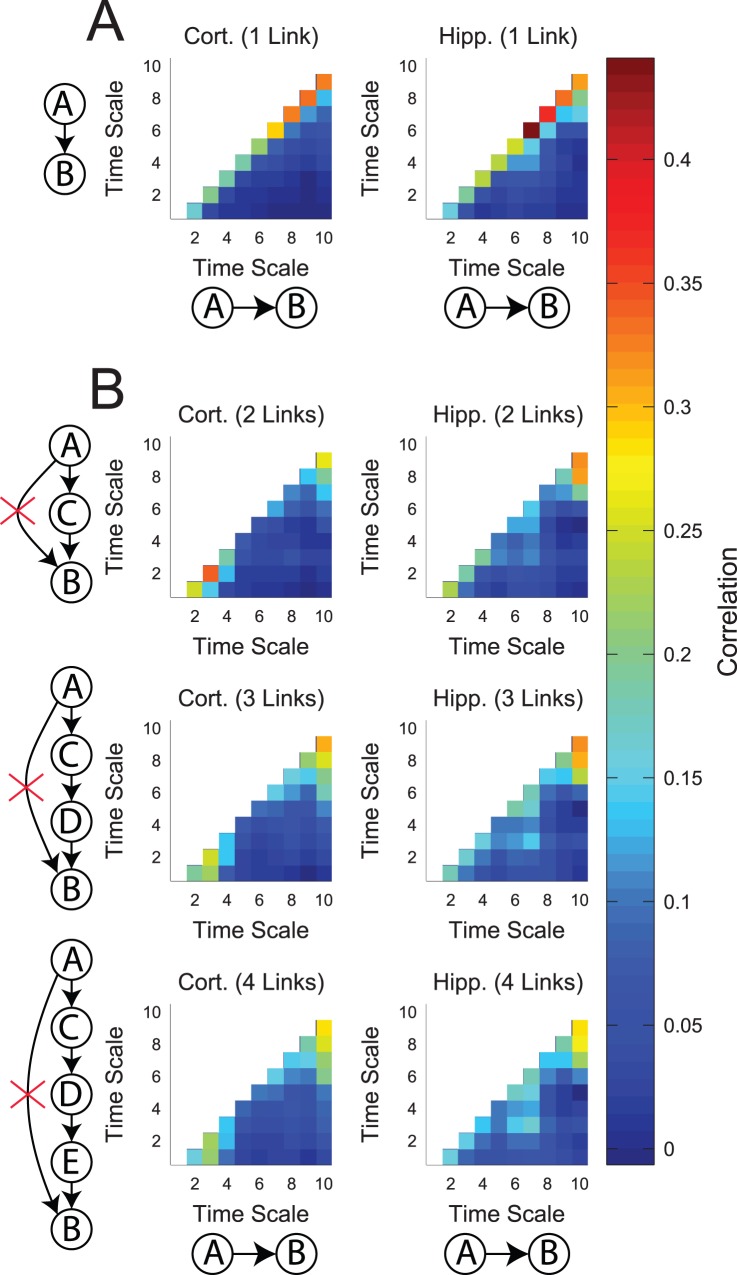
Long time scale connectivity was independent of short time scale connectivity. (**A**) Connectivity was most correlated at nearby time scales, but uncorrelated at distant time scales. Correlation was measured using all possible pairs of neurons where a connection (lack of connection) was assigned to 1 (0). (**B**) Chains of indirect connections at short time scales (time scale on vertical axes) were weakly correlated with direct long time scale connections (time scale on horizontal axes). Correlation was measured using all possible pairs of neurons where a connection or chain of indirect connections (lack of connection or chain) was assigned to 1 (0).

Before proceeding to examine the full effective connectivity networks, we addressed three issues involving individual neurons and connections. We first measured the correlation between the existence of connections at different time scales (Fig. 2 A). We might expect that neurons which communicate would do so at many time scales, which would lead to correlations between connectivity at different time scales. Alternatively, we might expect that connectivity would be segregated based on time scale, which would lead to anti-correlations between connectivity at different time scales. In fact, we found that connectivity was most strongly correlated at adjacent time scales and weakly correlated at distant time scales. High correlation at adjacent time scales is expected because the time scales overlapped (Fig. 1 E), but the weak correlation at distant time scales implies that short time scale connectivity was independent of long time scale connectivity.

Next, we examined the role chains of short time scale connections played in the existence of longer time scale connections. It might be suspected that long time scale connectivity was simply due to chains of short time scale connections. If this were true, then the network would not contain time scale dependent multiplex connections. Rather, it would contain only one type of connection. To investigate this concern, for each pair of neurons, we measured the correlation between the existence of a chain of significant connections and no direct connection at a short time scale with the existence of a direct significant connection at a longer time scale (Fig. 2 B). We found that chains of short time scale connections were correlated with longer time scale direct connections, but only weakly. Indeed, any effective connectivity analysis will be susceptible to false positive errors of this type (i.e. confusing A → C → B for A → B) [75]. However, using our multiple time scale analysis, we are able to evaluate this phenomenon and determine that if these false positive errors were occurring, they were occurring at a relatively low rate.

For the remaining network analyses, each of the effective connectivity networks was sub-sampled 500 times into smaller networks with 50 neurons and the resulting values for the sub-networks were averaged to obtain the final results for each data set (recording). Only a subset of the strongest connections corresponding to an average degree or number of connections per neuron (k) of 3 was retained in the sub-networks. This procedure was utilized to avoid biases based on network size and number of connections [76] (see *Materials and Methods*). Also, this method significantly reduced differences between hippocampal and cortical networks in terms of the number of neurons and the number of connections per neuron. Some of the data sets did not produce a sufficient number of significant interactions between neurons at one or more time scales. These data sets were excluded from further analysis (Fig. 1 D).

**Figure 3:**
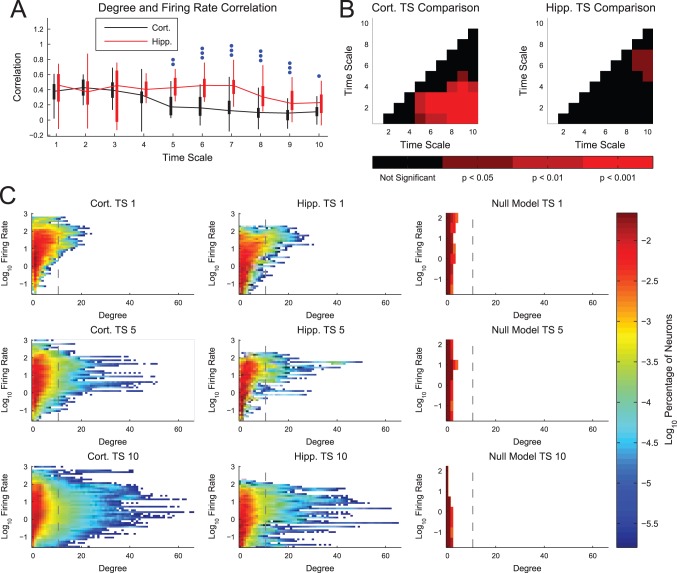
Firing rate and degree were correlated. (**A**) Neuron firing rate and degree were correlated, especially for short time scales. Hippocampal networks showed higher correlations than cortical networks for middle and long time scales. Box plots: minimum, 25^th^ percentile, median, 75^th^ percentile, maximum data set (recording). Differences between hippocampal and cortical networks were assessed with a multiple comparisons corrected Mann-Whitney Test (one dot: p<0.05, two dots: p<0.01, three dots: p<0.001). (**B**) The correlation between firing rate and degree generally decreased with time scale. Multiple comparison corrected Mann-Whitney Test p-values between different time scales for the same tissue type. (**C**) Density plots of neuron degrees and firing rates in sub-networks. Note that the real data contain many high degree neurons and the stronger correlation between degree and firing rate for short time scales (top row) in comparison to longer time scales. Also, note that the null model data contained very few non-zero degree neurons. This lack of connectivity in the null model implies that high degrees for high firing rate neurons are not the result of false-positive connections. Vertical line is the approximate degree threshold for hub classification.

To better understand the role firing rate played in our analysis, we next examined the correlation between neuron firing rate and degree. We found that firing rate and neuron degree were correlated in the sub-networks, especially for shorter time scales, with hippocampal networks showing higher correlation for middle and longer time scales (Fig. 3 A). Furthermore, we found that the correlation decreased with time scale, with the most significant changes in cortical networks (Fig. 3 B). Generally speaking, we found neurons with a wide range of firing rates possessed connections at all time scales, though the distribution was skewed towards high firing rate neurons having higher degree at short time scales (Fig. 3 C). To insure this correlation was not an artifact of the analysis, we examined the connectivity in a simple null model network. This model possessed 400 independent neurons with firing rates that spanned the firing rates seen in the real data. Each model neuron spiked randomly (Poisson) and was recorded for one hour. The null model produced less connectivity than expected by chance from multiple comparisons for all time scales and showed fewer neurons with 1 or more connections in comparison to the real data (Fig. 3 C).

From these results, we concluded that the correlation between neuron degree and firing rate is not an artifact of false-positive connections for high firing rate neurons. That said, for any type of analysis, it is true that decreased quantities of data will weaken the statistics and make an effect harder to detect. In our case, this general rule implies that lower firing rate neurons will have weaker statistics and it will be more difficult to detect effective connections involving low firing rate neurons. Therefore, it is possible that weak connections were missed (false-negatives) due to low firing rates. It is important to note that this issue is closely related to, but distinct from, issues surrounding structural and effective connectivity. If one neuron will influence the activity of another neuron (i.e. they are structurally connected), but the driving neuron never fires, the connection will not be detected (i.e. there will be no effective connection). Since effective connectivity is not simply the capacity to effect change, but also requires actually effecting change, it is correct to find that a neuron that never fires is not effectively connected to other neurons. If one were attempting to assess structural connectivity with effective connectivity, this scenario would be a concern. However, because we were only interested in the effective connectivity itself, this issue was not a concern for our analysis.

### Neuronal Hubs

**Figure 4:**
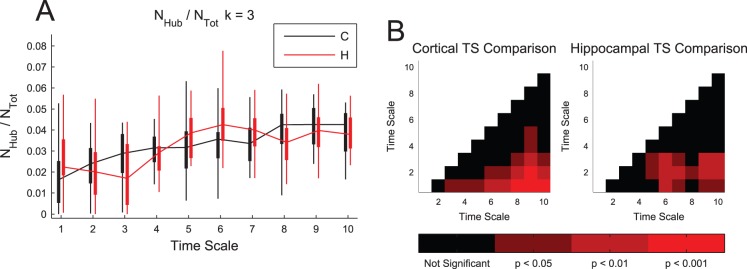
A small and consistent percentage of neurons were found to be hubs. (**A**) Most data sets exhibited a small, but significant number of hubs across all time scales. Note that the likelihood for a randomly connected neuron to be found to be a hub was set at 10^−4^ (see *Materials and Methods*), so these results indicate a roughly two order of magnitude increase in the number of hubs over a random network. Box plots: minimum, 25^th^ percentile, median, 75^th^ percentile, maximum data set (recording). No significant differences were found between hippocampal and cortical networks. (**B**) Multiple comparison corrected Mann-Whitney Test p-values between different time scales for the same tissue type. The number of hubs generally increased with time scale.

We next analyzed the proportion of the neurons in the network that were hubs (highly connected neurons). Given the fact that these neurons connect to many other neurons, we might expect they play a special role in the processing of information in the networks [72], [77]. The threshold for the number of connections required for a neuron to be classified as a hub was set using the likelihood (10^−4^) to find a highly connected neuron in a random network with the same number of neurons and connections (see *Materials and Methods*). In a sub-network of 50 neurons with average degree of 3, this threshold corresponded to 11 connections (Fig. 3 C). A small proportion of the neurons were found to be hubs in both types of tissue and at all time scales for most data sets (Fig. 4 A). The proportion of hubs expected by chance in a random network was set to 10^−4^, so we found roughly two orders of magnitude more hubs than would be expected by chance in a randomly connected network. Though it is not surprising to find more high degree neurons than would be expected in a random network, interestingly we did find that the proportion of neurons that were classified as hubs generally increased as time scale lengthened. To assess this phenomenon, we compared the distributions of hub percentages between different time scales for the same tissue (Fig. 4 B). Generally, we found that long time scales possessed a significantly larger percentage of hubs.

### Shared Hubs

**Figure 5:**
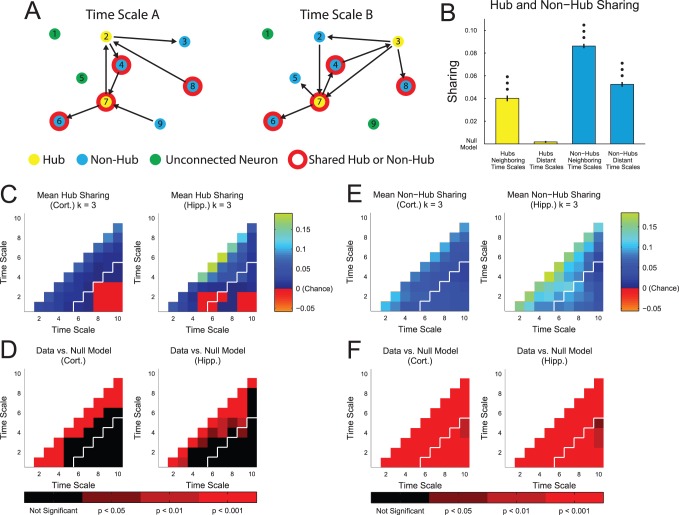
Hub sharing was limited to adjacent time scales. (**A**) We classified each neuron as a hub, non-hub, or unconnected neuron at each time scale. A neuron was considered to be a shared hub or shared non-hub for two time scales if its status as a hub or non-hub was consistent across those time scales. Hubs were defined using a degree threshold set by the likelihood to have a given number of connections in a random network (0.05 in this illustrative diagram and 10^−4^ in the full analysis). (**B**) We calculated the amount of hub and non-hub sharing (see *Materials and Methods*) for each pair of time scales and grouped the results into neighboring (4 or less) and distant (greater than 4) time scales. *We found that hubs were only shared at a significant level for neighboring time scales, while non-hubs were broadly shared across all time scales* (multiple comparisons correct Mann-Whitney Test (1, 2, and 3 dots: p<0.05, 0.01, and 0.001 respectively), error bar: standard error of the mean). For each data set, we subtracted the mean sharing values for 500 trials with neuron identities randomized and neuron hub, non-hub, or unconnected status held constant. This null model approximates the amount of sharing expected based only on the number of hubs, non-hubs, and unconnected neurons in the data set, as well as the effect of ignoring the multiplex properties of the networks and considering the time scales to be truly independent networks. We also calculated the mean sharing value of (**C**) hubs and (**E**) non-hubs across each pair of time scales for cortical and hippocampal networks. In (B), neighboring time scale pairs are up and to the left of the white line, while distant time scale pairs are down and to the right of the white line. (**D and F**) Finally, we calculated the multiple comparisons corrected Mann-Whitney Test p-values between sharing results from data and sharing results from the null model.

In addition to analyzing the proportion of neurons that were found to be hubs, we also compared the identity of the neurons that were found to be hubs at different time scales in the same data set (Fig. 5). We compared the status of each neuron as a participant with few connections in a network (non-hub) or as a participant with many connections in a network (hub) across different time scales (Fig. 5 A) by calculating the amount of hub and non-hub sharing (see *Materials and Methods*). In order to understand the importance of the hub and non-hub sharing results, for each data set, we subtracted from the original sharing value the mean sharing from 500 trials of a null model. The null model consisted of the same neurons as the original data, including their status as hubs, non-hubs, or unconnected neurons. However, for each trial in the null model, the identities of the neurons were randomized. Thus, positive sharing values implied more sharing than expected by chance and negative sharing values implied less sharing than expected by chance. This allowed us to evaluate which results were due to time scale and neuron specific behavior in the networks (i.e. uniquely multiplex network properties) and which results were due simply to the fact that the vast majority of connected neurons were non-hubs. Surprisingly, we found that hubs tended to be shared across nearby time scales, while non-hubs tended to be shared widely across all time scales (Fig. 5 B). In other words, *the neurons that were found to be hubs at one time scale were not usually found to be hubs at other distant time scales. Conversely, neurons that were found to be participants in networks, but which had few connections (non-hubs), tended to also participate in networks at other time scales.* In addition, we calculated the sharing values for each pair of time scales and both tissue types (Fig. 5 C–F). We found that hub sharing was actually below the level expected by the null model for many distant time scales (Fig. 5 C), though the differences between the sharing values from the data and from the null model were not significant for these time scale pairs (Fig. 5 D). This lack of significance may be due to the large number of comparisons for which we had to correct, as well as the small number of hubs. In fact, when we performed the same analysis with a lower degree threshold for hub classification – thereby increasing the number of neurons that were classified as hubs – some of the negative sharing results for hub neurons were found to be significant for cortical networks (S2 and S3 D and E Figs). It should be noted that, because adjacent time scales overlap (Fig. 1 E), we might expect elevated hub and non-hub sharing across adjacent time scales. The fact that we find this result implies that networks at adjacent time scales are similar and supports the validity of the analysis method. Also, elevated sharing in nearby, though not adjacent, time scales cannot be explained merely by the overlap in adjacent time scales (Fig. 5 C–F).

### Connection Distance

**Figure 6:**
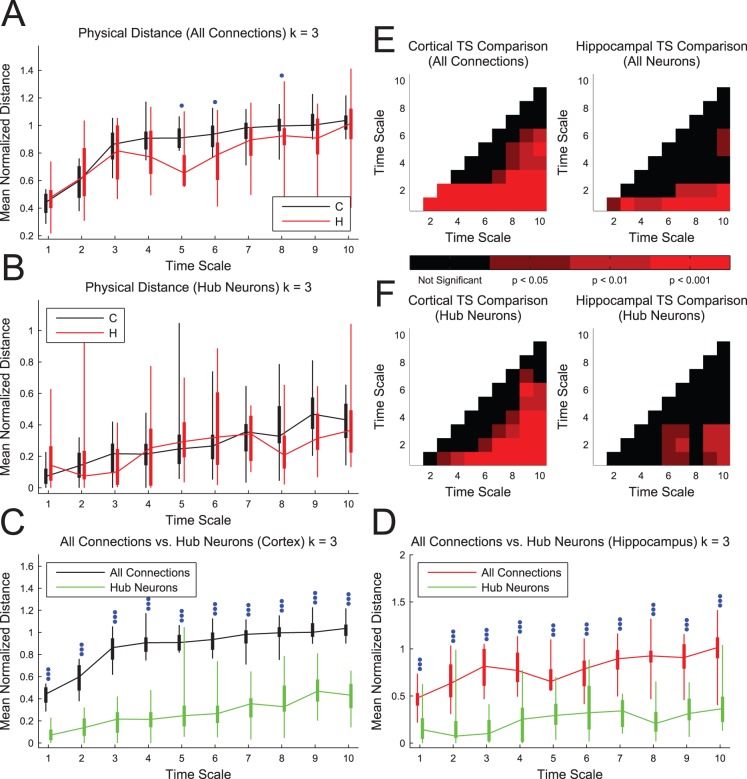
Physical distance between connected neurons increased with time scale and hubs were closely spaced. (**A**) The mean physical connection distance was calculated as the ratio of the mean physical distance between effectively connected neurons in the network to the mean physical distance between all possible pairs of neurons in the sub-network. Cortical networks were found to be significantly larger for several time scales. (**B**) The mean physical distance between hubs was calculated as the ratio of the mean physical distance between hubs (connected or not connected) to the mean physical distance between all possible pairs of neurons in the sub-network. No significant differences between hippocampal and cortical networks were observed. (**C and D**) The hubs were significantly more closely spaced than the average connected pair. Box plots: minimum, 25^th^ percentile, median, 75^th^ percentile, maximum data set (recording). Differences between hippocampal and cortical networks were assessed with a multiple comparisons corrected Mann-Whitney Test (one dot: p<0.05, two dots: p<0.01, three dots: p<0.001). (**E and F**) Multiple comparisons corrected Mann-Whitney Test p-values between different time scales for the same tissue type for connection distances (E) and hub distances (F). Note that cortical and hippocampal network connections tend to be significantly longer in time scales 3 to 10 in comparison to time scales 1 and 2. Also, note that hub distances generally increase with time scale.

Next, we examined the relationship between physical distance and time scale dependent connectivity (Fig. 6). We compared the average distance between effectively connected neurons at each time scale to the average distance of all possible connections (Fig. 6 A). We found that cortical connections were significantly longer for several time scales. Furthermore, we found that the average connection length was typically shorter than the average length of all possible connections (i.e. mean connection distances less than 1), indicating that the actual network is smaller than the network of all possible connections. When we compared these distances across time scales for the same tissue (Fig. 6 E), we found that the average connection distances were significantly shorter for the first two time scales in both types of tissue. This result seems reasonable given the assumption that, when given more time, information is more likely to spread to distant neurons. We also compared the average distance between hub neurons at each time scale to the average distance of all possible connections (Fig. 6 B) and we did not find significant differences between tissue types. We found that the distance between hubs generally increased with time scale (Fig. 6 F). Finally, we found that the distance between hubs was significantly smaller than the average connection distance in both tissue types and for all time scales (Fig. 6 C and D).

### Topology Results

To investigate the time scale dependent topology of the networks, we applied two network topology measures to the cortical and hippocampal effective connectivity networks using programs from the Brain Connectivity Toolbox [78].

**Figure 7:**
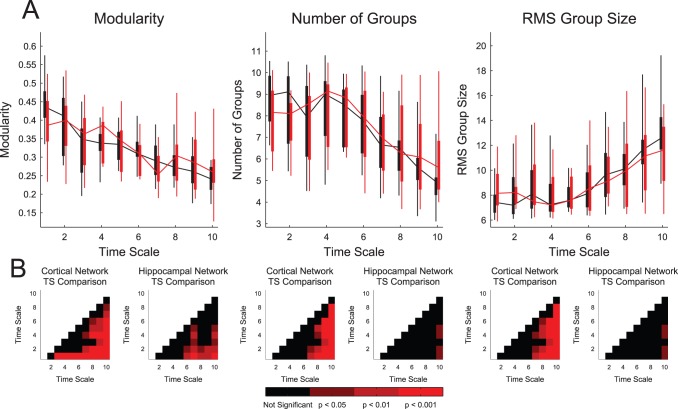
Network modularity decreased with time scale. (**A**) Network modularity and the number of modules generally decreased with time scale, while the size of the modules generally increased with time scale. Box plots: minimum, 25^th^ percentile, median, 75^th^ percentile, maximum data set (recording). No significant differences between hippocampal and cortical networks were observed. (**B**) Multiple comparisons corrected Mann-Whitney Test p-values across different time scales for identical tissue types. Note that the changes with time scale in (A) were generally significant, especially for cortical networks.

First, we measured the modularity of the networks (Fig. 7). Modularity measures the degree to which the network can be separated into non-overlapping groups. Most interestingly, we found that the modularity tended to decrease with time scale (Fig. 7 A) and these changes were generally significant (Fig. 7 B). As we might expect, we also found fewer and larger modules as the time scale increased (Fig. 7 A). For all modularity results, we found no significant differences between tissue types at individual time scales, though the time scale dependent behavior of the cortical networks tended to be more significant across different time scales in comparison to the hippocampal networks. The general result that, at short time scales, the neurons formed networks with many well defined and small modules, while at longer time scales the neurons formed networks with few large and poorly defined modules supports the hypothesis that short time scale interactions are part of distinct modules while longer time scale interactions connect those modules.

**Figure 8:**
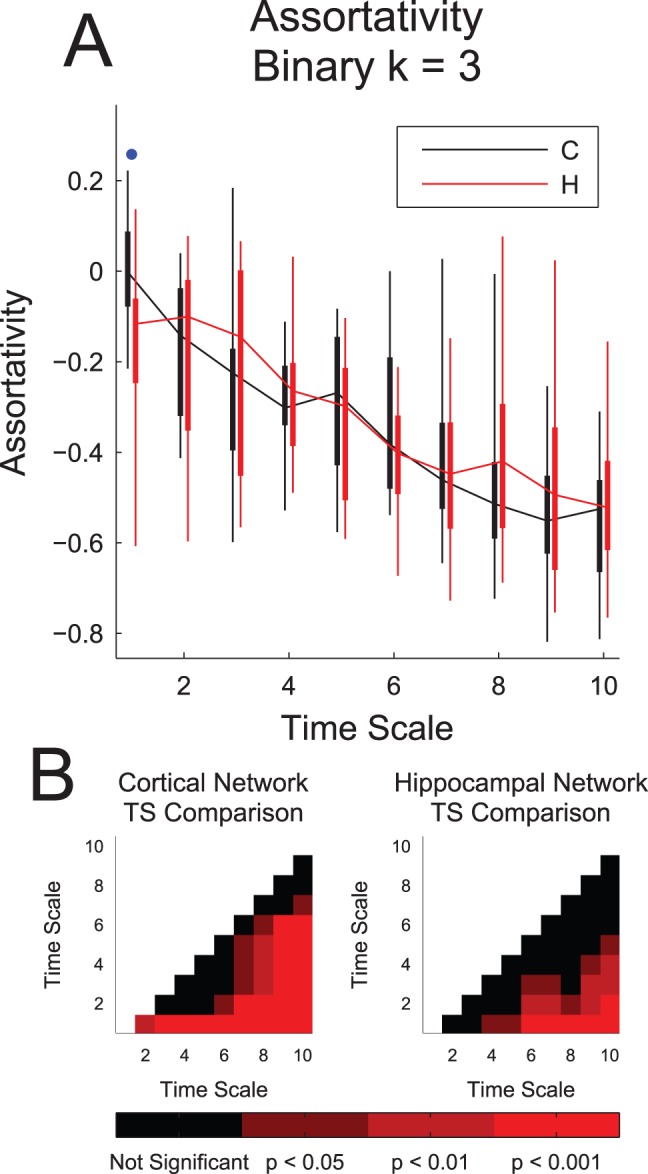
Network assortativity decreased with time scale. (**A**) Network assortativity generally decreased (disassortativity generally increased) with time scale. Note the significantly higher assortativity for cortical networks at time scale 1 (interaction delays of 0.05 ms to 3 ms), and that the networks were generally disassortative. Box plots: minimum, 25^th^ percentile, median, 75^th^ percentile, maximum data set (recording). Differences between hippocampal and cortical networks were assessed with a multiple comparisons corrected Mann-Whitney Test (one dot: p<0.05, two dots: p<0.01, three dots: p<0.001). (**B**) Multiple comparisons corrected Mann-Whitney Test p-values across different time scales for identical tissue types. Note that the decreasing behavior in (A) was generally significant.

Second, we measured the assortativity of the networks (Fig. 8). We used the form of assortativity that measures the correlation between the number of outgoing connections of neurons at the start of connections and the number of incoming connections of neurons at the end of connections. If this correlation is positive, the network is said to be “assortative,” but if the correlation is negative, the network is said to be “disassortative.” Thus, in assortative networks, neurons with many outgoing connections tend to connect to neurons with many incoming connections. In disassortative networks, neurons with many outgoing connections tend to connect to neurons with few incoming connections. We found that the networks were generally disassortative. Also, we found significant differences between the tissue types only at the shortest time scale, where the cortical networks were found to be more assortative (Fig. 8 A). Both tissues showed significant decreases in assortativity (increases in disassortativity) as the time scale lengthened (Fig. 8 B). This implies that, at long time scales, neurons with many outgoing connections connected to neurons with few incoming connections, while neurons with few outgoing connections connected to neurons with many incoming connections.

## Discussion

### Multiplex Networks

*Our analysis represents the first systematic examination of time scale dependent multiplex networks of individual neurons and it indicates that these time scale dependent networks potentially differ by function and brain region.* Previous *in vitro* studies have shown changes in connectivity with time scale [46]–[48], but those works considered each time scale as an isolated network. By examining the correlation between connections at different time scales, as well as the hub and non-hub sharing properties of the networks, we examined the connectivity of individual neurons across time scales. Therefore, for the first time, we evaluated uniquely multiplex network properties in networks of individual neurons.

The result that hub neurons tended to be isolated at specific time scales, while non-hubs were not is especially compelling given recent research on fault tolerance in multiplex networks [4], [11], [14], [15]. Our result indicates that if a neuron that operates as a hub at one time scale were removed from the network, the impact would be reduced to networks at other time scales because it would be less likely that the removed neuron operated as a hub at other time scales. Therefore, we hypothesize that this arrangement of connectivity increases fault tolerance in comparison to a system in which the hub functionality is concentrated in a few neurons across all time scales. In a future experiment, the role of hub neurons in network fault tolerance could be studied if it were possible to identify and selectively remove hub (high degree) and non-hub (low degree) neurons. We predict that if a hub neuron which operates as a hub at only one time scale is selectively removed from the network, the effect on the behavior of the network at another time scale would be reduced in comparison to the removal of a neuron that operates as a hub at that time scale.

In addition to possible implications for fault tolerance, the low correlation between connections and the lack of sharing of hubs across distant time scales that we found also has implications for recent studies of correlations between node degree and connectivity in other types of networks (see [79] and the applications therein). In contrast to many of those studies, our results point to similarity only in terms of non-hubs across different types of connections (time scales).

### Hubs

In this paper we have presented compelling evidence that hub neurons are time scale specific and non-hub neurons are not time scale specific. Hub neurons are of special interest because they are connected to many other neurons and, therefore, possibly play a unique role in network operation. Neural hubs and their importance have been studied in brain regions [69], [77], [80], [81], but we know of only three previous analyses that examined hubs on the scale of individual neurons [72], [82], [83]. We feel our results compliment these previous results in that, while we were unable to identify hub neurons by type, we were able to examine network behavior at a wide range of time scales.

Bonifazi et. al. found hubs to be GABAergic interneurons in developing hippocampal acute slices [72], [82] (see [84] for a recent discussion of GABAergic hub neurons). In comparison to our analysis, the work by Bonifazi et. al. was different in that it employed cross-correlation to identify functional connections, the recordings were performed and analyzed at only one time scale (∼50 ms, calcium imaging, see Fig. 1 E), and acute hippocampal slices from developing rats and mice were studied instead of organotypic slice cultures from mice. Though cross-correlation was used by Bonifazi et. al. to identify hubs, they were also able to show that altering the activity of the hub neurons affected overall network behavior. In the future, it will be interesting to see if the effective hub neurons we found at the ∼50 ms time scale in hippocampal networks are also GABAergic interneurons, as well as whether the hubs we found at other time scales and in the cortex are also GABAergic interneurons.

Quilichini et. al. found that hippocamposeptal neurons play a special, hub-like, role in controlling gamma oscillations at the onset of ictal-like events in acute slices [83]. Unlike our analysis or the work by Bonifazi et. al., the researchers in this study used patch-clamp to record from individual neurons and compared their behavior to the behavior of network-wide large-scale gamma-frequency oscillations. Because the researchers in this study defined “hub” in terms of initiation and control over gamma oscillations, we were unable to directly determine if the hub neurons we found in the gamma frequency time scales (TS 3–5) were hippocamposeptal neurons.

Our result that hub neurons function as hubs at specific time scales indicates that*hub neurons may be intimately related with time scale specific phenomena in the brain.* It has been hypothesized that interactions at different time scales (e.g. oscillations at different frequencies) are crucial for information integration in the brain [32]–[37], so the hub neurons identified in our work may play a unique role in neural networks. In future experiments, we would like to investigate the role the hub neurons play in a wide range of network behavior. For instance, do hub neurons at a specific time scale initiate or maintain network bursts? What role do hub neurons play in sensory coding? Do hub neurons at a specific time scale encode certain features of the stimulus? If the hub neurons do encode information about a stimulus, do they do so synergistically or redundantly? Finally, it may be possible to better connect the role of hubs in network behavior if it were possible to identify the type(s) of neuron(s) that operate as hubs in the network.

### Time Scales

*Importantly, the results of our analysis indicate that individual neurons interact over time scales ranging from sub-millisecond (gap junction, synapse) to seconds (hemodynamic response)*. Only the short time scale interactions (up to TS 4, see Fig. 1 E) could possibly be due to monosynaptic physical connectivity. Furthermore, we found only a weak correlation between the existence of chains of short time scale connections and the existence of long time scale connections. This indicates that the long time scale connections cannot be solely attributed to chains of short time scale connections. In the future, we hope to better understand the origins of the longer time scale interactions and how connectivity at the different time scales is related to other neurological phenomena.

Regarding our specific results, *we found that several features of the effective connectivity networks were time scale dependent*. For instance, we found changes in physical distance between effectively connected neurons, changes in network topology measures, and time scale dependencies among hub/non-hub neurons and connectivity. Previous studies have examined temporal multiplexing in brain signals [32]–[39], but we know of only two studies to date that have focused on different time scales in effective connectivity networks at the single neuron level [46]–[48].

In a previous study of the same data sets [46], [48], we analyzed time scale dependent functional connectivity instead of time scale dependent effective connectivity, as was analyzed herein. In that work, we applied wavelet transforms to the cross-correlations between the neurons and grouped the resulting significant connections in frequency ranges. That analysis found time scale dependent connectivity properties, but it treated each separate time scale as an independent network. In the present analysis, we examined time scale dependent connectivity properties, but we also examined the hub properties of neurons and connectivity correlation across various time scales. Thus, though the previous study implicitly involved multiplex networks, only the present analysis examined truly multiplex network properties. Also, it should be noted that the definitions of “time scale” applied in our present study and this previous work were significantly different (wavelet transform versus delay) and that the functional and effective connectivity of these networks were significantly different.

Matsuda et. al. used TE to examine the effective connectivity in dissociated cultures [47]. Similarly to our analysis, Matsuda et. al. used different bin sizes to probe different time scales. However, their binning structure did not isolate interactions at a certain time scale by including a minimum delay and they only focused on time scales shorter than 100 ms. Also, they used only four data sets and did not attempt to determine which TE results were statistically significant. Furthermore, the effective connectivity networks in their dissociated cultures were probably less similar to *in vivo* networks in comparison to the organotypic cultures we utilized. Finally, like the work by Ito et. al., Matsuda et. al. treated each time scale as an independent network and did not examine uniquely multiplex properties.

Our analysis demonstrates that effective connectivity networks among individual neurons vary with time scale in interesting and potentially complex ways. Some of our results seem quite reasonable, such as the increasing physical distance between effectively connected neurons with increased time scale and the decrease in modularity with time scale. In the future, more work must be done to understand how the changes in effective network connectivity relate to different neural phenomena at different time scales.

### Network Topology

A great deal of research has focused on whether brain networks are scale-free or small-world (see [41], [65] for examples using fMRI, [46], [48], [70], [72], [85] for examples involving individual neurons, and [3] for a review). We did not directly assess whether the networks studied in this paper possessed scale-free degree distributions. However, we did observe significantly more high degree neurons than would be expected in a random network. So, while we cannot determine if the networks we examined were scale-free, small-world, hierarchical, etc., we can determine they were most likely not random and that they possessed heavy tailed degree distributions.

By applying several topology measures to the effective connectivity networks, we were able to produce a picture of the networks that change with time scale. For instance, *we found that the networks become significantly less modular as time scale increased*. In other words, at long time scales the networks contained a few large and poorly defined groups (modules), while at shorter time scales, the networks contained many small and well differentiated groups. This result supports the general hypothesis that the brain consists of hierarchical modules [86], [87], though our results also imply that as the module size increases, so too does the time scale at which the module processes information. Furthermore, this result may have significant implications for the time scales involved in population coding [88]. In the future, we hope to investigate the relationship between the time scale dependent modules and sensory coding. Do all the neurons in one module code a certain feature of the stimulus, and, if so, is it time scale specific?

Next, *we found that the networks became more disassortative as the time scale increased*. This implies that, at short time scales, the connections were more evenly distributed with regards to directionality. However, at long time scales, high out-degree neurons tended to connect to low in-degree neurons, while low out-degree neurons tended to connect to high in-degree neurons. This result may have implications for information flow, the role of hub neurons at long versus short time scales, and the organizational mechanisms in these networks [89].

Beyond the topological qualities of the observed networks mentioned above, it would also be interesting to investigate possible mechanisms that could lead to such topologies through the development of the networks. Numerous mechanisms for network formation have been introduced [20], [90], [91]. In the future, the topological results presented in this analysis could contribute to the delineation of the mechanisms that are biologically realizable in neural networks.

### Transfer Entropy and Other Measures of Connectivity

TE has previously been used in many neuroscience applications [27], [45], [47], [50]–[59], [61]–[64]. However, *no one, to the best of our knowledge, has used TE with different binning and delays to systematically study interactions at multiple isolated time scales ranging from sub-millisecond to seconds.* The precise values of bin size, delay, and state structure we chose (Fig. 1 E) are not vital to the analysis. We simply chose those values to span a wide range of time scales in logarithmically nearly-equal delay windows. If so desired, a researcher could change the time scales to better suit their research topic. Overall, the *results of this analysis demonstrate that TE can be used successfully as a tool to examine effective interactions between single neurons over many time scales.* Importantly, the recently proposed BRAIN Initiative seeks to record all neural activity across complete neural systems [92]. The analysis presented herein represents a novel method to analyze the data produced by this important initiative.

By comparing neuron degree and firing rate (Fig. 3), *we found that neuron degree and firing rate were correlated*. The lack of connectivity in a null model implied that there were likely few false-positive connections in the analysis based on firing rate, but there may have been false-negative results for low firing rate neurons due to poor statistics. We feel it is also important to highlight the role that the distinction between structural and effective connectivity plays in this issue. In short, even if neurons are structurally connected, if they do not fire or fire very infrequently, then they are not effectively connected. This perceived inability to detect structural connectivity with effective connectivity is actually a misalignment between the definitions of these two types of connectivity. This point is especially important for studies that seek to relate structural connectivity with effective and functional connectivity [22]–[31].

In addition to TE, researchers have also used Granger Causality [93]–[95] in similar neural systems (see [96]–[101] for examples and [102] for a discussion of Granger Causality and TE graphs in neuroscience). Also, researchers have used Dynamic Causal Modeling to measure effective connectivity in neural systems (see [103]–[105] as examples). We chose to use TE because, in its basic form, it is model independent and can capture non-linear interactions, unlike Granger Causality and Dynamic Causal Modeling. Furthermore, our analysis utilized binary spike data, which is better suited for TE than Granger Causality or Dynamic Causal Modeling. Finally, the ability of TE to render interactions in terms of bits – a general unit of information – allows for straightforward comparisons between networks and graphs.

Besides TE, Granger Causality, and Dynamic Causal Modeling, researchers have also used cross-correlation (CC) and mutual information to infer functional connectivity [35], [50], [72], [106]. Both CC and mutual information are measures of functional connectivity because they measure statistical dependencies between the activities of neural sources without taking into account the effect or causal influence of one neural source on another.

We chose to analyze TE networks instead of CC networks for five primary reasons. First, unlike CC, TE measures interactions in terms of bits which are a general unit that can be easily compared across different connections and systems. Second, TE is a measure of effective connectivity, whereas CC is a measure of functional connectivity. Therefore, TE is able to capture how the activity of the transmitting neuron affects the activity of the receiving neuron, while CC is only able to measure how their activities are correlated. Third, TE is able to measure non-linear interactions, while CC is not. Fourth, though it was not a direct goal in this analysis, TE has been shown to be superior at inferring physical connectivity [50], [63]. Fifth, TE’s information theoretic nature means that it will easily allow the analysis to be extended to include multivariate information measures, which is a future goal of our research.

### Limitations of this Study

Perhaps the most noticeable potential limitation of this analysis is the fact that it was performed using organotypic cultures [107], [108]. Although organotypic cultures have been widely used in research [109], [110], these cultures have been shown to possess several differences in comparison to the *in vivo* system using both mice and rats. Such differences *in vitro* include additional synaptic connectivity [111], [112], decreased ease of LTP induction [113], changes in protein expression [114], increased excitability [112], [115], and changes in cellular organization in mice [116].

Despite these issues, the overall structure and electrical activity of cortico-hippocampal organotypic cultures have been shown to essentially match the *in vivo* system [111], [113], [117]. Furthermore, it has been shown that interneurons in organotypic cultures are physiologically and morphologically identical to interneurons *in vivo* [118], cortical layer structure and cell migration are preserved in postnatal organotypic cultures (as were used in this analysis) in rats [119], and that intracortical connection structure is preserved in organotypic cultures when sub-cortical regions are preserved in culturing (as was done in this analysis) [120]–[122].

Based on these previous studies, we concluded that organotypic cultures represent a useful model system for intact *in vivo* neural systems. Therefore, we believe our results are highly relevant for the field given the strength of the preparation used, the power of the analysis, and the novelty of the results themselves. Furthermore, at this time,*it would not have been technologically possible to achieve the same level of spatial and temporal recording resolution and the same number of recorded neurons in vivo.* While some *in vivo* recording methods are capable of recording from hundreds of neurons, these methods demand trade-offs in terms of temporal or spatial resolution. For instance, *in vivo* calcium imaging allows for the simultaneous recording of up to approximately 1000 neurons, but the temporal resolution for these recordings is significantly less (tens of ms) than we achieved in our recordings (50 µs) [123]–[125]. Recording methods with lower temporal resolution would have been unable to capture the short time scale interactions that we observed. Furthermore, *in vivo* electrophysiological recording methods that employ planar arrays or shank electrodes are capable of recording hundreds of neurons with high temporal resolution, but these recording methods possess limited spatial resolution in comparison to our array (inter-electrode spacing of 60 µm) due to larger inter-electrode spacing in arrays (e.g. 400 µm in Utah arrays (Blackrock Microsystems)) and larger spacing between shanks (e.g. 250 µm in [126]). Recording methods with larger inter-electrode spacing would have been less likely to detect short time scale interactions, since those interactions were found to occur primarily between closely spaced neurons (Fig. 6 A). Therefore, our use of organotypic cultures and a high density, high temporal resolution multi-electrode array permitted a dramatic improvement in the quality of the data, which improved the strength of the analysis.

Our recording method possessed several distinct features that were advantageous especially during the developmental stages of this method. Still, other recording methods could be used with this TE analysis method to investigate other phenomena. For instance, the use of *in vivo* calcium imagining, while lacking the temporal resolution to capture short time scale connections, would more easily facilitate the gathering of additional information about the neurons involved in the networks (e.g. cell type, cell layer, etc.) and would more easily allow for direct cell stimulation or inhibition via optogenetic techniques [127]. Furthermore, *in vivo* studies could investigate the relationship between time scale dependent connectivity and phenomena that can only be studied *in vivo*, such as behavior and sensory coding. We feel these types of analyses could produce novel insights into time scale dependent networks in the brain and we plan to pursue them in the future.

## Materials and Methods

A general overview of the analysis is presented in S4 Fig.

### Ethics Statement

All neural tissue samples from animals were prepared according to guidelines from the National Institutes of Health and all animal procedures were approved by the Indiana University Animal Care and Use Committee (Protocol: 12–015) as well as the Animal Care and Use Committee at the University of California, Santa Cruz (Protocol: Litka1105).

### Gathering the raw data, pre-processing, and spike sorting

The raw spiking data utilized in this analysis are fully described elsewhere [48]. Briefly, cortico-hippocampal organotypic cultures were produced using postnatal day 6 Black 6 mouse pups (wild-type C57BL/6 from Charles River) following the protocol described in [107]. The mice were anesthetized in an ice bath prior to decapitation and brain removal. Each culture was recorded after 2 to 4 weeks. After culturing, spontaneous activity was recorded from each slice using a custom-made 512-electrode array system [128]. The array contained 5 µm diameter flat electrodes arranged in a hexagonal lattice with an inter-electrode distance of 60 µm. In this arrangement, the total recording area of the array was approximately a 1 mm by 2 mm rectangle. Each recording was performed such that either the hippocampus or the cortex was centered on the array. Action potentials (spikes) were then detected and spike-sorted using a well-established method [128]. Briefly, points in time where voltage traces exceeded 8 standard deviations calculated over a 5 second window of the voltage trace were marked as potential spikes on a given electrode. A portion of the voltage trace for the given electrode and the 6 adjacent electrodes were then utilized as spike waveforms. These waveforms were then projected into a five dimensional principal component space. Clustering of these points (each of which represents one potential spike) was then performed using a mixture of Gaussian models with maximum likelihood estimation. Duplicate neurons and neurons with many refractory period violations were then excluded from further analysis (see [128] for additional details). After spike sorting, neurons with less than 100 spikes in the 60 minute recording (firing rate <0.028 Hz) were removed from the analysis. Then, the resulting spike times were used in the remainder of the analysis.

**Figure 9:**
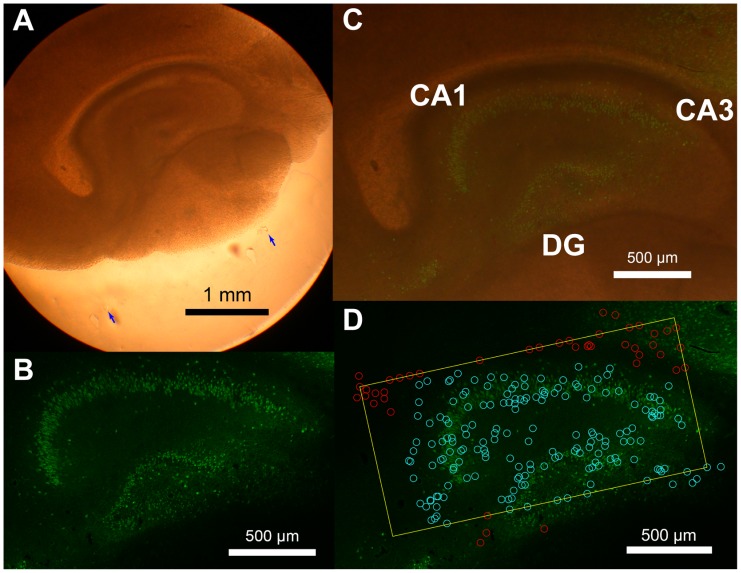
Hippocampal structures were preserved throughout culturing. Photographs of cortico-hippocampal organotypic cultures. (**A**) A bright field image of an example organotypic culture at DIV1. The hippocampal structure is visible without staining. Blue arrows indicate the location of the edge of the recording array. (**B**) NeuN staining of the culture after data taking and tissue fixation at DIV16. There are missing neurons in CA3 as consistent with a previous report [111], but the overall layer structure is well conserved. (**C**) Overlaid photograph of A and B. Positions and dimensions of the hippocampal structures are well conserved during the incubation period. (**D**) Overlaid photograph of B, the outline of the array (yellow rectangle), and the estimated locations of the recorded neurons. Light blue circles are manually identified hippocampal neurons and red circles are neurons recorded outside the hippocampal structure. Locations of the recorded neurons match with the granule cell layer and the cell body layer. For complete details on culture preparation, see [48].

After electrophysiological recording, six example cultures were stained with NeuN to check for differentiation between hippocampal and cortical tissue at the point of recording (Fig. 9) (see [48] for complete staining details). The results of this staining procedure indicated that hippocampal structure was well maintained from DIV1 throughout culturing. In addition to the six example cultures that underwent staining, all live tissue from hippocampal recordings was also imaged pre- and post-recording using light microscopy. These images were aligned and overlaid with cell positions from the electrode array and the neurons were manually sorted as falling within hippocampal or cortical tissue. We did not attempt to identify from which region of the hippocampus or layer of the cortex each neuron originated because the differentiation between these tissue regions was difficult to observe under light microscopy for all cultures (see *Discussion – Limitations of this Study*). Cortical recordings did not require this procedure as the cortex was large enough to cover the entire array. In both cases, cell positions on the array were measured by fitting a two-dimensional Gaussian distribution to the signal strengths of each neuron on the electrodes on which they were recorded.

### Burst Statistics

Network bursts of various types were observed in all of the data sets we analyzed [129], [130] (Fig. 1 A and S1 Fig). To calculate burst statistics for our data, we used the general procedure described by Wagenaar et al. [129] to define individual neuron bursts, with one altered parameter. First, we detected neuron bursts by finding groups of at least 4 consecutive spikes with inter-spike intervals (ISIs) less than one-eighth (one-fourth in Wagenaar et. al.) of the average ISI for that neuron. Second, based on observations of the data and the appearance of large bursts that involved many neurons in the network, we felt it was necessary to also measure network bursts. So, we referred to a group of overlapping neuron bursts that contained at least 10% of the neurons in the network as a network burst. Note that we did not require that at least 10% of the neurons be bursting at the same time. Rather, we only required that there was no period during the network burst where no neurons were bursting and that at least 10% of the neurons burst at some point during the network burst. To insure that these bursts did not bias our analysis, we generated model data that contained bursts, but no other interactions between the neurons. In these model data, we found roughly the number of connections expected by chance in random data, while in the actual data we found significantly more connections.

### Transfer Entropy Analysis

Transfer Entropy (TE) was introduced by Schreiber to measure the influence of one time series (call it I) on another time series (call it J) [49]. In our case, I and J were binary spike trains for neurons I and J that contained 0 for the time bins when the neuron did not spike and 1 for the time bins when the neuron did spike. In its most basic form, the TE from I to J is given by:
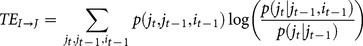
(1)


By definition, the TE from neuron I to neuron I is zero, so no self connections existed in our analysis. The probabilities in Eqn. (1) are typically measured by counting all the instances of the possible combinations of spikes and no spikes in the j_t_, j_t-1_, and i_t-1_ bins for all time bins. Note that this process requires the assumption that the time series are stationary and a sufficiently long recording is used to adequately approximate the probability distribution. Given the length of our recordings (1 hour), the fact that spontaneous activity was recorded, and that there are limited combinations of possible states (2^3^) due to the binary nature of our time series, we feel TE can be used to generate meaningful results from our data.

Generally speaking, TE measures the information gained about the state of the target time series (j_t_) when the past state of a transmitting time series (i_t-1_) is known, beyond the information provided by the past state of the target time series (j_t-1_) alone. TE has been used in several neuroscience applications [27], [45], [47], [50]–[56], [59], [60], [63], [64]. In its most basic form, TE is only able to capture interactions with delays falling within adjacent time bins. For instance, if it were the case that neuron J tended to spike ten time bins after neuron I spiked, the spikes would be so far apart that no interaction would be present in adjacent time bins and the basic TE expressed in Eqn. (1) would not detect an interaction. It would be possible to capture this ten time bin interaction by simply rebinning the data to combine multiple time bins together. However, this procedure could retain some short time scale interactions, yielding a TE result that would be polluted with short time scale interactions.

In order to measure interactions over many *isolated* time scales, we included a delay between the past states of the neurons (i_t-d_ instead of i_t-1_ and j_t-d_ instead of j_t-1_) and we systematically varied the bin size across ten time scales. The delay was time scale dependent to allow for the shortest time scale to capture the shortest interactions allowed by the resolution of the recordings. Also, we combined the i_t-d_ and j_t-d_ states with their preceding time bin (i_t-d-1_ and j_t-d-1_) in such a way that a spike in either or both time bins yielded an overall state of 1 and no spikes in either time bins yielded an overall state of 0. We denote these new states as i’_t-d_ and j’_t-d_. This leads to a slightly altered TE expression:
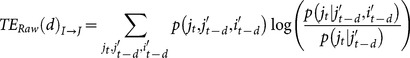
(2)


We also calculated the normalized TE by dividing the raw TE by the entropy of the J spike train. Doing so changes the interpretation of the TE from the amount of information being transmitted to the percentage of the target neuron’s entropy that can be accounted for by the transmitting neuron. This modified version of the TE is given by:
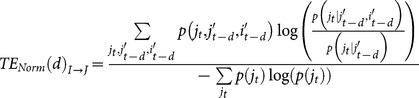
(3)


The binary connections throughout the analysis were taken to be 1 whenever the raw TE value was significant (see below for the significance testing method) for a given pair of neurons and 0 when it was not.

**Figure 10:**
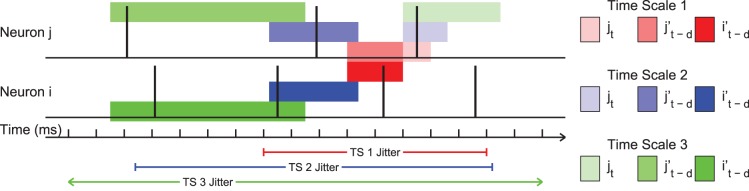
Binning structure for short time scales on example spike trains. Note that the time scales overlapped to some degree to capture interactions with all delays and that time scales greater than 1 possessed delays to prevent short time scale interactions from influencing long time scale measurements.

We chose to create ten time scales at which to analyze the isolated TE values. We created the time scales in such a way that they logarithmically spanned a large range of neurologically relevant time scales. The precise values of the bin sizes and delays for each time scale are given in Table 1. To more clearly communicate the binning structure and delay windows, the three smallest time scales are overlaid on an example spike train in Fig. 10. The interaction time scales (delay windows) for the ten time scales are presented in Fig. 1 E along with several neural phenomena and common measurement methods for easy comparison. Overall, this method allowed us to systematically examine interactions between individual neurons *in vitro* over time scales ranging from sub-milliseconds to seconds.

**Table 1:** Time scales.

Time Scale	Bin Size (ms)	d	Delay Window (bins)	Delay Window (ms)	Jitter Window (ms)
1	1	0	0–3	0.05–3	7
2	1.6	1	1–4	1.6–6.4	11.2
3	3.5	1	1–4	3.5–14	24.5
4	7.5	1	1–4	7.5–30	52.5
5	16.15	1	1–4	16.15–64.6	113.05
6	34.8	1	1–4	34.8–139.2	243.6
7	75	1	1–4	75–300	525
8	161.6	1	1–4	161.6–646.4	1131.2
9	348.1	1	1–4	348.1–1392.4	2436.7
10	750	1	1–4	750–3000	5250

As the time scale increased, the bin sizes logarithmically increased. The overall state structure with regards to the bins was identical for time scales 2 through 10. Time scale 1 possessed a delay of 0 (d = 0) in order to capture interactions at the smallest resolution of the recordings (0.05 ms).

Wibral et al. recently proposed an alternative method for measuring delayed interactions with TE [131]. In their method there is no delay for the history of the J time series (i.e. j’_t-d_ becomes j_t-1_ in Eqn. (2)). As they elegantly show, this alternative method is well suited to detect the delay which produces the maximum TE. Both the alternative method [63] and methods similar to the one employed in this analysis [52], [54], [55], [132] have been used previously. Despite the advantages of the Wibral et. al. method, we were forced to utilize a method that included a delay in the J time series in order to isolate interactions in separate time scales. In comparison, the analysis by Wibral et. al. sought to find the delay that produced the maximum TE, so it did not allow a delay in the J time series to produce a fair comparison of TE values. Fortunately, our analysis did not involve the comparison of TE values at different delays, rather it sought to find significant TE values at isolated time scales. Therefore, we feel that the concerns correctly expressed by Wibral et. al. regarding the inclusion of a delay in the J time series do not apply to our method.

**Figure 11:**
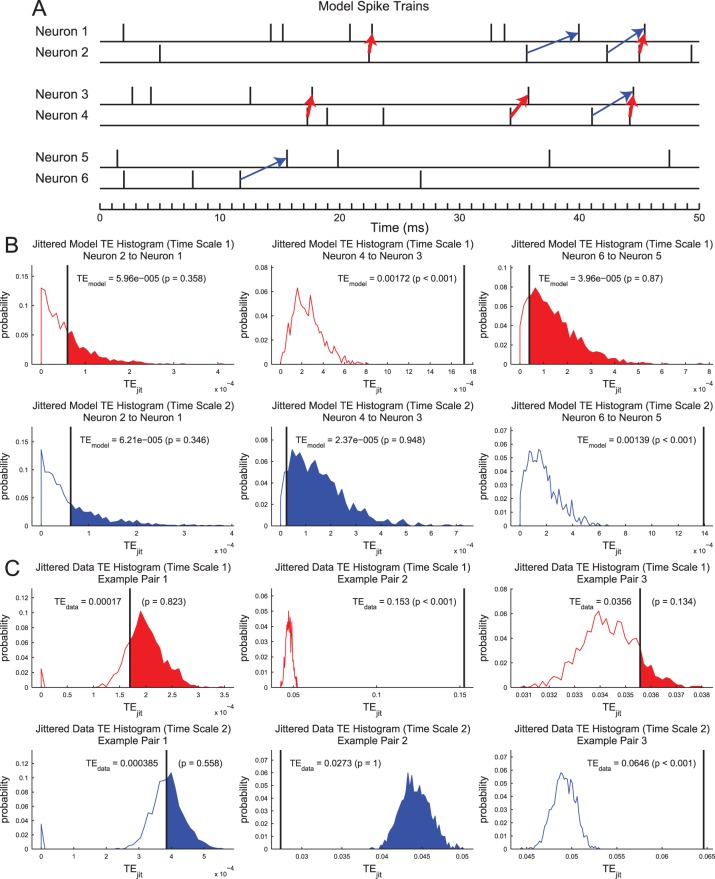
The TE analysis method isolated time scale specific interactions. (**A**) Spike trains for 6 model neurons. All neurons spike randomly (Poisson) at 100 Hz and were recorded for 60 seconds. (**B**) Jittered TE histograms for the model neurons for the two shortest time scales. Neurons 1 and 2: Independent spiking. TE results are not significant for either time scale. Neurons 3 and 4∶5% of neuron 3 spikes were moved to follow 1.5 ms after neuron 4 spikes. A significant TE result is found for the first time scale (0.05 ms to 3 ms), but not for the second time scale (1.6 ms to 6.4 ms). Neurons 5 and 6∶5% of neuron 5 spikes were moved to follow 4 ms after neuron 6 spikes. A significant TE result is found for the second time scale (1.6 ms to 6.4 ms), but not for the first time scale (0.05 ms to 3 ms). (**C**) Example jittered TE histograms from real data that show similar features to model neuron pairs.

In order to assess which TE values were statistically significant, we employed a Monte Carlo approach to generate a distribution of TE values from randomized data. To generate the randomized data, we jittered the I spike train using a uniform distribution with a width of seven bins centered on the original location of each spike. The firing rate of the I spike train was preserved in the jittering process, as were the number of i’_t-d_ = 1 states. We only jittered the I spike train in order to preserve interactions in the J spike train. We used a uniform distribution for the jittering to prevent spikes from being jittered in front of or behind the j_t_ state of the J spike train. Doing so prevented strong connections from neuron J to I from obscuring weaker connections from neuron I to J. The jittering and TE calculation procedure was performed 5000 times for each pair of neurons. The p-value for the original TE measurement was then calculated as the proportion of the jittered data sets that produced TE values greater than the value from the original data. Examples of jittered TE distributions can be seen for three pairs of simple model neurons in Fig. 11 B and corresponding examples pairs from the real data can be seen in Fig. 11 C.

**Figure 12:**
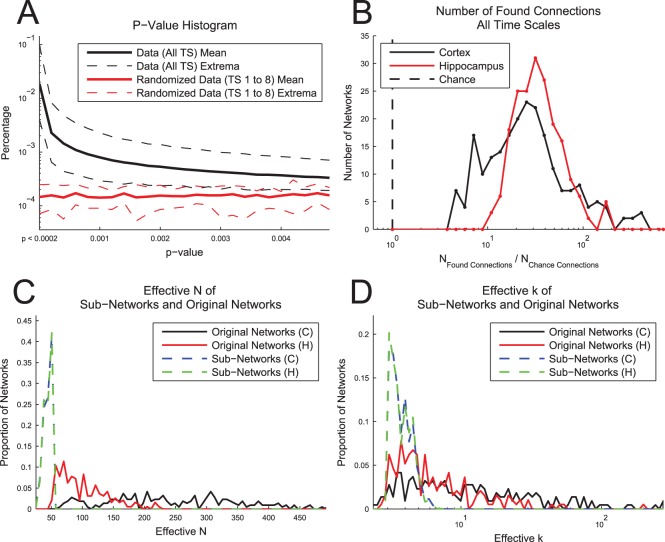
Connectivity statistics. (**A**) TE p-value histogram for real and randomized data. Real data show many more pairs of neurons with low p-values compared to randomized data. In this analysis, the p-value threshold was set at less than 0.001. The extrema correspond to the time scale with the largest or smallest percentage value for a given p-value. (**B**) Number of found connections. The number of connections found in each network was at least 4 times larger than expected by chance, with most networks containing 10 to 100 times more connections than expected by chance. (**C**) Effective N values. The effective N for each network is the number of connected nodes in the network. The mean ± STD in the original data (sub-networks) was 241±103 (42±7) for cortical networks and 128±54 (42±7) for hippocampal networks. (**D**) Effective k values. The effective k for each network is the average number of connections (degree) per neuron. The mean ± STD in the original data (sub-networks) was 22±30 (3.7±0.7) for cortical networks and 11±18 (3.7±0.8) for hippocampal networks. Note that the sub-network procedure significantly reduced the variability of the effective N and k values, as well as the differences in effective N and k between tissue types.

We chose to set a p-value threshold of p<0.001 for the TE results in this analysis. Pairs of neurons with p-values above this threshold were removed from the remainder of the analysis. We chose this threshold after examining results from randomized data (Fig. 12 A). In order to test the effects of network bursts [129], [130] on the analysis, we generated 8 randomized data sets (4 from hippocampal recordings and 4 from cortical recordings) that contained random Poisson firing for each neuron during network bursts (firing rate matched to bursts in real data) and no spikes for all other times. Here, we identified network bursts using a manually set threshold for total network activity in 100 ms bins. Many more connections were found to have low p-values in the real data in comparison to these randomized data, especially for p<0.001. We only compared results for the first 8 time scales because the longer time scales were on the same scale as the network bursts that were retained in the randomized data. In order to ensure that the false-positive connections did not affect the overall analysis, we repeated the entire analysis using p<0.0002 as the p-value threshold. Other than fewer viable data sets (see *Topology Analysis – Sub-Networks*) and the resulting degradation of the statistics used to evaluate differences between tissue types, we found no appreciable differences in the results.

Though the p-value threshold we imposed was small (p<0.001), many of the networks had many possible connections. So, to insure that the connections we found to be significant were not merely the product of multiple measurements, we calculated the ratio between the number of connections found in the analysis and the expected number of connections found by chance (Fig. 12 B). In a network with N neurons, there are N*(N –1) possible directed connections. So, by chance we would expect approximately p*N*(N –1) spurious connections, where p is the p-value threshold of 0.001. We found that the data sets with the smallest ratio still possessed roughly 4 times more connections than expected by chance. The majority of the data sets possessed between 10 and 100 times more connections than expected by chance. Therefore, we feel it is unlikely that spurious false-positive connections from multiple measurements biased our analysis to a significant degree.

### Topology Analysis – Sub-Networks

Before analyzing the full networks, we applied a sub-network creation routine to avoid biases associated with network size (N) and average degree or number of connections per neuron (k). After the initial network construction, we found that each data set and time scale possessed greatly varied numbers of neurons and connections (Fig. 12 C and D). It has been shown that network topology measures are N and k dependent [76]. To compensate for this effect and to ensure that measured differences between networks are not simply due to N and k, we created 500 sub-networks for each data set and time scale with nearly matching N (target: 50) and k (target: 3) values (detailed procedure below). It was not possible to create sub-networks with precisely identical N and k values, but we feel the wide range of N and k values present in the original data, as well as the large differences between hippocampal and cortical networks in terms of N and k values, were significantly reduced using the sub-network routine (Fig. 12 C and D). We feel that this reduction in variability reduced biases associated with N and k to the highest degree possible at this time. Each sub-network was then analyzed and the resulting values were averaged over all sub-networks to obtain an unbiased result for each data set and time scale.

The sub-network creation procedure was as follows: First, the neurons were randomly divided into a group of 50 neurons for the sub-network and a pool of unselected neurons. Second, if any of the 50 sub-network neurons were unconnected with the other neurons in the sub-network, an unconnected neuron was replaced with a randomly selected neuron from the pool of other neurons. This process was repeated until all neurons in the group of 50 had at least one connection or until no adequate substitution could be found, in which case, the algorithm would restart. If the algorithm could not succeed 500 times in 10^8^ attempts, the data set was deemed unviable for that time scale and it was removed from the analysis. We felt 500 sub-networks of 50 neurons were sufficient to completely sample all of the networks because, even in our largest network of approximately 650 neurons, each neuron would be randomly selected for the sub-network at least approximately 25 times. If we had been using larger data sets, it would be necessary to use larger sub-networks or more sub-networks. Third, the k value was enforce by retaining only the (k*N)/2 strongest connections, as defined by the normalized TE (Eqn. 3).

We found it was necessary to replace unconnected neurons in the sub-network creation routine to avoid creating sub-networks with many unconnected neurons. If allowed to remain in the sub-network, unconnected neurons would effectively decrease N and increase k because unconnected nodes were ignored in the topology analyses, so replacing unconnected neurons allowed us to more closely match the N and k values between sub-networks. It should be noted that it was not always possible to produce sub-networks with 50 connected neurons after setting the k value using the strongest TE values. Still, we feel this algorithm was the best solution available to the problem of N and k dependent topology measures because it substantially reduced the variance in the N and k values across the networks (Fig. 12 C and D).

### Topology Analysis – Hubs

Once the sub-networks were created, hub neurons were identified by their degree. Using a binomial distribution, we estimated the likelihood to observe a neuron with a given degree in a randomly connected network with an identical number of neurons and connections. If the likelihood to observe a given degree was less than 10^−4^, we marked neurons with that degree as hubs. We used a binomial distribution to ease processing time and because the number of connections was so small that replacement effects could be ignored. We identified hubs based on total degree (number of incoming and outgoing connections). For example, for sub-networks that contained 50 neurons and had k = 3 average connections per neuron, the degree threshold for being considered a hub was 11 total connections. We also applied thresholds of 10^−2^ (8 connections) and 10^−3^ (9 connections) to the data (S2 and S3 Figs.).

Once the hubs were identified, we calculated the percentage of the neurons that were found to be hubs for each sub-network. We then averaged these results for all 500 sub-networks. This produced a value for the percentage of neurons that were found to be hubs for each data set and time scale. We then plotted the distribution of the average values for all hippocampal and cortical data sets for each time scale (Fig. 4) and performed a multiple comparisons corrected Mann-Whitney Test on the two distributions to determine if they differed significantly.

Other authors have defined hubs using different features of nodes in the network [78], [133]. We chose to use degree because it is simple and straightforward, but we also feel that other methods of identifying hubs may provide useful and interesting results.

To compare the hubs across different time scales, we examined each neuron’s status in the network of a data set at different time scales (Fig. 5). While the neurons in the network were identical at different time scales, their status as connected or unconnected, as well as their status as hubs or non-hubs could change with time scale. We wished to calculate the degree to which the neurons that were hubs at one time scale were also hubs at another time scale (hub sharing), as well as the degree to which the neurons that were non-hubs at one time scale were also non-hubs at another time scale (non-hub sharing). This task was complicated by the use of sub-networks. To calculate the amount of hub sharing, we utilized the following procedure: First, we calculated the ratio of the number of times each neuron was found to be a hub in a sub-network over the number of times each neuron was found in a sub-network. This ratio was referred to as the hub score for the i^th^ neuron and u^th^ time scale:
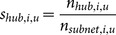
(4)


If a neuron was never found to be in a sub-network, its hub score was set to zero. Second, we calculated the product of the corresponding hub scores between one pair of time scales for one data set. Third, we calculated the average of the hub score products for which either the multiplicand or multiplier was non-zero. This final result was the hub sharing value for one data set and one pair of time scales:
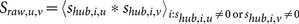
(5)


Using this method, if, for instance, one data set possessed two neurons that were always found to be hubs at two different time scales, it would yield a hub sharing value of 1. If, for instance, one data set possessed two neurons that were always found to be hubs at one time scale and two different neurons that were always found to be hubs at a different time scale, it would yield a hub sharing value of 0.

Fourth, in order to differentiate the effects of the distribution of hub scores from the influence of time scale and neuron identities, we constructed a null model by calculating the hub sharing values for randomized trials. Each trial retained the original hub scores for each neuron, but the identities of the neurons were randomized:
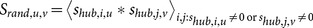
(6)where the sequence of j neurons is a randomized version of the sequence of the i neurons. In this way, the hub sharing values for the randomized trials produced results that estimated the hub sharing values in the case where only the distribution of hub scores is relevant. Fifth, 500 randomized trials were performed, averaged for each data set and time scale, and subtracted from the original hub sharing value. This produced the final hub sharing value:



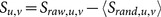
(7)By including the randomized trial comparison, the hub sharing value described the degree of hub sharing above or below what would be expected by chance in the case where the only relevant feature is the distribution of hub scores.

Sixth, we averaged the hub sharing values across all data sets for time scales that were at most four steps apart (“neighboring time scales”) and for time scales that were more than four steps apart (“distant time scales”) (Fig. 5 B). We also averaged the hub sharing values for a given tissue type and pair of time scales (Fig. 5 C and E). Finally, we compared the distribution of hub sharing results to the distribution of hub sharing results for the randomized trials (also corrected by the mean of the sharing results for the randomized trials) to assess when the hub sharing results differed significantly from the null model (Fig. 5 B, D, and F).

The procedure to compare non-hubs across different time scales was identical to the procedure for hubs with the exception that we calculated a non-hub score by calculating the ratio of the number of times each neuron was found to be a non-hub in a sub-network over the number of times each neuron was found in a sub-network:
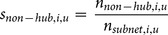
(8)


### Topology Analysis – Connection Distance

Using the physical location of each neuron on the array, we were able to examine the physical distance between effectively connected neurons and hubs. To do so, we calculated the ratio of the mean physical distance between all connected neurons in a sub-network and the mean physics distance between all hub neurons (connected or not) to the mean physical distance between all possible connections in the same sub-network. We then averaged these values over all sub-networks to obtain the average normalized network size and hub distance for a single data set and time scale. Finally, we plotted the distributions of these average results for hippocampal and cortical data at all time scales (Fig. 6). We compared the distributions of normalized distance values for connections and hubs for the two tissue types using a multiple comparisons corrected Mann-Whitney Test. We also compared the hub distances to the connection distances, as well as the hub distances and connection distances across different time scales using a multiple comparisons corrected Mann-Whitney Test.

### Topology Analysis – Topology Measures

In addition to analyzing the hubs and physical connection distances, we applied three topology measures to the networks using software from the Brain Connectivity Toolbox [78]. Specifically, we used modularity and assortativity.

Modularity measures the degree to which the network could be divided into separate modules [134], [135]. A high modularity corresponded to well divided, non-overlapping groups. In addition to the modularity, we also counted the number of modules found and root mean square of the size of the modules. Unlike assortativity, the modularity calculation was stochastic. Therefore, we calculated the modularity, the number of modules, and the root mean square of the module sizes 10 times for each sub-network and averaged the resulting values to obtain mean values for each sub-network we analyzed.

Assortativity measures the correlation between the degrees of nodes at the ends of connections [136]. In our case, we used the correlation between the out-degree of nodes at the starting point of connections and the in-degree of nodes at the end point of connections. A high positive assortativity implies that the high out-degree nodes connect to high in-degree nodes, while low out-degree nodes connect to low in-degree nodes. On the other hand, a large negative assortativity (disassortativity) implies that the high out-degree nodes connect to low in-degree nodes, while low out-degree nodes connect to high in-degree nodes.

After calculating the topology measures for the sub-networks and averaging those results to obtain a single value for each data set and time scale, we compared the distributions of the topology measures for hippocampal and cortical networks using a multiple comparisons corrected Mann-Whitney Test (Figs. 7 and 8). We also compared the distributions of topology values for identical tissue types across different time scales using a multiple comparisons corrected Mann-Whitney Test.

### Correction for Multiple Comparisons

Given the large number of comparisons performed in this analysis, it was necessary to correct for the effects of multiple comparisons. We chose to use False Discovery Rate (FDR) control to limit the likelihood of spurious false-positive results. Specifically, we utilized the algorithm introduced by Benjamini and Yekutieli [137] as a modification of the earlier algorithm introduced by Benjamini and Hochberg [138]. We implemented the algorithm using software created by Groppe et al. [139]. This method has been shown to work correctly for dependent and independent measurements. We felt that it would not have been appropriate to use a familywise error rate correction (e.g. Bonferroni) for three primary reasons [139]. First, this study was generally exploratory in nature and we anticipated that noteworthy results would likely be distributed across many comparisons. Second, given the relatively few culture recordings, we anticipated that the individual comparisons would be relatively weak. Third, FDR does apply a conservative correction in the case of few significant comparisons (see Fig. 8 for a noteworthy example).

We applied the correction to the Mann-Whitney test results for each unique type of measurement that was performed across different time scales or different pairs of time scales. For instance, we applied this correction to the Mann-Whitney p-values for the hub sharing results for the cortical networks for all pairs of time scales. We did not use FDR to correct for false-positive results in the TE measurements for individual pairs of neurons due to the extremely large number of comparisons that were performed, as well as the large number of connections found in comparison to the number of connections expected by chance (Fig. 12 B).

## Supporting Information

S1 Fig **Burst Statistics.** Neuron burst were detected using the algorithm described in [123], with one altered parameter. Briefly, a neuron was said to burst if 4 consecutive spikes had inter-spike intervals (ISIs) of less than one-eighth the average ISI for that neuron. A network burst was defined as any grouping of overlapping neuron bursts that contained at least 10% of the neurons in the network. 10% of the neurons in the network were not required to be bursting at the same time. **(A)** Firing rates for neurons during bursts are roughly two orders of magnitude larger than outside of bursts. **(B)** Most bursts only involved a small subset of the neurons, but a significant proportion of the bursts involved many neurons (so called “network bursts”). **(C)** Most neurons in each data set burst and most of the bursting neurons participated in network bursts. **(D and E)** Network burst durations were typically between 1 and 10 seconds, though significantly shorter and longer bursts were observed. Also, most network bursts were separated by about 10 seconds, though significantly shorter and longer burst separations were observed. Black box plots: cortical data sets. Red box plots: hippocampal data sets. Box plot: minimum, 25^th^ percentile, median (cyan dot), 75^th^ percentile, maximum value.
(EPS)

S2 Fig **Hub Results with Alternative Degree Threshold (10^−2^).** Identical analyses to those presented in Figs. 2 and 3, except with lowered degree threshold. For N = 50 and k = 3, the threshold of 10^−4^ (manuscript) implies a degree threshold of 11, while the threshold of 10^−2^ implies a degree threshold of 8. **(A, D–G)** Fig. 5. **(B and C)** Fig. 4. For this threshold, we found no significant changes in percentage of neurons found to be hubs across different time scales **(B and C)**. Notice that hub sharing values were significantly below the level in the null model for distant time scales in cortical networks **(D and E)**, though not in hippocampal networks **(F and G)**. This indicates that in cortical networks, hub functionality was not just randomly distributed across distant time scales, but rather, hub functionality was distributed into separate groups.
(EPS)

S3 Fig **Hub Results with Alternative Degree Threshold (10^−3^).** Identical analyses to those presented in Figs. 2 and 3, except with lowered degree threshold. For N = 50 and k = 3, the threshold of 10^−4^ (manuscript) implies a degree threshold of 11, while the threshold of 10^−3^ implies a degree threshold of 9. **(A, D–G)** Fig. 5. **(B and C)** Fig. 4. For this threshold, we found few significant changes in percentage of neurons found to be hubs across different time scales **(B and C)**. Notice that hub sharing values were significantly below the level in the null model for distant time scales in cortical networks **(D and E)**, though not in hippocampal networks **(F and G)**. This indicates that in cortical networks, hub functionality was not just randomly distributed across distant time scales, but rather, hub functionality was distributed into separate groups.
(EPS)

S4 Fig **Analysis overview.** 1 (Blue): Raw data were gathered from organotypic cultures using the 512-electrode array. The data were then pre-processed and spike sorted. 2 (Purple): The neuron firing rates, network sizes, and burst statistics were calculated. 3 (Orange): The data were rebinned to the appropriate time scale. The TE was then calculated. 4 (Brown): Step 3 was repeated 5,000 times using jittered data. 5 (Magenta): The p-value for each normalized TE result was calculated as the percentage of the jittered data sets that produced normalized TE values greater than or equal to the result from the data. Any connections above the p-value threshold were removed from the analysis. In this analysis, the p-value threshold was set at p<0.001. Significant connections were then labeled as binary connections in the networks. 6 (Dark Blue): The relationships between firing rate and degree, as well as the correlation between connectivity at different time scales were analyzed. 7 (Yellow): To compensate for differences in N and k between data sets, sub-networks of 50 neurons were randomly created from the binary networks and the average number of connections per neuron (k) was set by retaining only the strongest connections. Topology measures were applied to the sub-networks. 8 (Dark Green): The sub-network results were averaged and analyzed.
(EPS)

## References

[1] Friston KJ (2011) Functional and effective connectivity: a review. Brain Connectivity 1. 10.1089/brain.2011.0008 22432952

[2] Scholvinck ML, Leopold DA, Brookes MJ, Khader PH (2013) The contribution of electrophysiology to functional connectivity mapping. NeuroImage 80:297–306. 23587686 10.1016/j.neuroimage.2013.04.010 PMC4206447

[3] Bullmore E, Sporns O (2009) Complex brain networks: graph theoretical analysis of structural and function systems. Nature Reviews Neuroscience 10. 10.1038/nrn2575 19190637

[4] Buldyrev SV, Parshani R, Paul G, Stanley HE, Havlin S (2010) Catastrophic cascade of failures in interdependent networks. Nature 464:1025–1028. 20393559 10.1038/nature08932

[5] Kurant M, Thiran P (2006) Layered complex networks. Physical Review Letters 96. 10.1103/PhysRevLett.96.138701 16712049

[6] Bianconi G (2013) Statistical mechanics of multiplex networks: entropy and overlap. Physical Review E 87. 10.1103/PhysRevE.87.062806 23848728

[7] Bagrow JP, Lehmann S, Ahn YY (2011) Robustness and modular structure in networks. arXiv: 1102.5085v1.

[8] Gao J, Buldyrev SV, Stanley HE, Havlin S (2011) Networks formed from interdependent networks. Nature Physics 8:40–48.

[9] Cardillo A, Gomez-Gardenes J, Zanin M, Romance M, Papo D, et al**.** (2013) Emergence of network features from multiplexity. Scientific Reports 3. 10.1038/srep01344 PMC3583169 23446838

[10] Gomez-Gardenes J, Reinares I, Arenas A, Floria LM (2012) Evolution of cooperation in multiplex networks. Scientific Reports 2. 10.1038/srep00620 PMC3431544 22943006

[11] Brummitt CD, Lee KM, Goh KI (2012) Multiplexity-facilitated cascades in networks. Physical Review E 85. 10.1103/PhysRevE.85.045102 22680529

[12] D’Agostino G, Scala A, editors (2014) Networks of networks: the last frontier of complexity. Switzerland: Springer International.

[13] Goni J, Heuvel MPvd, Avena-Koenigsberger A, Mendizabel NVd, Betzel RF, et al (2014) Resting-brain functional connectivity predicted by analytic measures of network communication. Proceedings of the National Academy of Sciences 111:833–838. 10.1073/pnas.1315529111 PMC3896172 24379387

[14] Boccaletti S, Bianconi G, Criado R, del Genio CI, Gomez-Gardenes J, et al**.** (2014) The structure and dynamics of multilayer networks. Physical Reports 10.1016/j.physrep.2014.07.001: In Press. 10.1016/j.physrep.2014.07.001 PMC7332224 32834429

[15] Baxter GJ, Dorogovtsev SN, Goltsev AV, Mendes JFF (2014) Avalanches in multiplex and interdependent networks. In: D’Agostino G, Scala A, editors. Networks of networks: the last frontier of complexity. Switzerland: Springer International.

[16] Sporns O (2007) Brain connectivity. Scholarpedia 2.

[17] Sporns O (2013) Structure and function of complex brain networks. Dialogues in Clinical Neuroscience 15:247–262. 24174898 10.31887/DCNS.2013.15.3/osporns PMC3811098

[18] van den Heuvel MP, Hulshoff Pol HE (2010) Exploring the brain network: A review on resting-state fMRI functional connectivity. European Neuropsychopharmacology 20:519–534. 20471808 10.1016/j.euroneuro.2010.03.008

[19] Feldt S, Bonifazi P, Cossart R (2011) Dissecting functional connectivity of neuronal microcircuits: experimental and theoretical insights. Trends in Neuroscience 34:225–236. 10.1016/j.tins.2011.02.007 21459463

[20] Sporns O, Chialvo DR, Kaiser M, Hilgetag CC (2004) Organization, development and function of complex brain networks. Trends in Cognitive Sciences 8:418–425. 15350243 10.1016/j.tics.2004.07.008

[21] Horwitz B (2003) The elusive concept of brain connectivity. NeuroImage 19:466–470. 12814595 10.1016/s1053-8119(03)00112-5

[22] Rykhlevskaia E, Gratton G, Fabiani M (2008) Combining structural and functional neuroimaging data for studying brain connectivity: a review. Psychophysiology 45:173–187. 17995910 10.1111/j.1469-8986.2007.00621.x

[23] Damoiseaux JS, Greicius MD (2009) Greater than the sum of its parts: a review of studies combining structural connectivity and resting-state functional connectivity. Brain Structure and Function 213:525–533. 19565262 10.1007/s00429-009-0208-6

[24] Honey CJ, Sporns O, Cammoun L, Gigandet X, Thiran JP, et al (2009) Predicting human resting-state functional connectivity from structural connectivity. Proceedings of the National Academy of Sciences 106:2035–2040. 10.1073/pnas.0811168106 PMC2634800 19188601

[25] Horn A, Ostwald D, Reisert M, Blankenburg F (2013) The structural-functional connectome and the default network of the human brain. NeuroImage 10.1016/j.neuroimage.2013.09.069. 10.1016/j.neuroimage.2013.09.069 24099851

[26] O’Reilly JX, Croxson PL, Jbabdi S, Sallet J, Noonan MP, et al (2013) Causal effect of disconnection lesions on interhemispheric functional connectivity in rhesus monkeys. Proceedings of the National Academy of Sciences 110:13982–13987. 10.1073/pnas.1305062110 PMC3752223 23924609

[27] Honey CJ, Kotter R, Breakspear M, Sporns O (2007) Network structure of cerebral cortex shapes functional connectivity on multiple time scales. Proceedings of the National Academy of Sciences 104:10240–10245. 10.1073/pnas.0701519104 PMC1891224 17548818

[28] Kispersky T, Gutierrez GJ, Marder E (2011) Functional connectivity in a rhythmic inhibitory circuit using Granger causality. Neural Systems and Circuits 1. 10.1186/2042-1001-1-9 PMC3314404 22330428

[29] Gerhard F, Kispersky T, Gutierrez GJ, Marder E, Kramer M, et al**.** (2013) Successful reconstruction of a physiological circuit with known connectivity from spiking activity alone. PLOS Computational Biology 9. 10.1371/journal.pcbi.1003138 PMC3708849 23874181

[30] Izquierdo EJ, Beer RD (2013) Connecting a connectome to behavior: an ensemble of neuroanatomical models of C. elegans klinotaxis. PLOS Computational Biology 9. 10.1371/journal.pcbi.1002890 PMC3567170 23408877

[31] Wang Z, Chen LM, Negyessy L, Friedman RM, Mishra A, et al (2013) The relationship of anatomical and functional connectivity to resting-state connectivity in primate somatosensory cortex. Neuron 78:1116–1126. 23791200 10.1016/j.neuron.2013.04.023 PMC3723346

[32] Varela F, Lachaux JP, Rodriguez E, Martinerie J (2001) The brainweb: phase synchronization and large-scale integration. Nature Reviews Neuroscience 2. 10.1038/35067550 11283746

[33] Canolty RT, Knight RT (2010) The functional role of cross-frequency coupling. Trends in Cognitive Sciences 14:506–515. 20932795 10.1016/j.tics.2010.09.001 PMC3359652

[34] Panzeri S, Brunel N, Logothetis NK, Kayser C (2010) Sensory neural codes using multiplexed temporal scales. Trends in Neuroscience 33:111–120. 10.1016/j.tins.2009.12.001 20045201

[35] Salinas E, Sejnowski TJ (2001) Correlated neuronal activity and the flow of neural information. Nature Reviews Neuroscience 2:539–550. 11483997 10.1038/35086012 PMC2868968

[36] Gire DH, Restrepo D, Sejnowski TJ, Greer C, Carlos JAD, et al (2013) Temporal processing in the olfactory system: can we see a smell? Neuron 78:416–432. 23664611 10.1016/j.neuron.2013.04.033 PMC3694266

[37] Akam T, Kullmann DM (2014) Oscillatory multiplexing of population codes for selective communication in the mammalian brain. Nature Reviews Neuroscience 15:111–122. 24434912 10.1038/nrn3668 PMC4724886

[38] Buzsaki G (2006) Rhythms of the Brain. New York: Oxford University Press.

[39] Kiebel SJ, Daunizeau J, Friston KJ (2008) A hierarchy of time-scales and the brain PLOS Computational Biology 4. 10.1371/journal.pcbi.1000209 PMC2568860 19008936

[40] Koenig T, Studer D, Hubl D, Melie L, Strik WK (2005) Brain connectivity at different time-scales measured with EEG. Philosophical Transactions of the Royal Society B 360:1015–1024. 10.1098/rstb.2005.1649 PMC1854932 16087445

[41] Achard S, Salvador R, Whitcher B, Suckling J, Bullmore E (2006) A resilient, low-frequency, small-world human brain functional network with highly connected association cortical hubs. Journal of Neuroscience 26:63–72. 16399673 10.1523/JNEUROSCI.3874-05.2006 PMC6674299

[42] Douw L, van Dellen E, de Groot M, Heimans JJ, Klein M, et al**.** (2010) Epilepsy is related to theta band brain connectivity and network topology in brain tumor patients. BMC Neuroscience 11. 10.1186/1471-2202-11-103 PMC2936439 20731854

[43] Mantini D, Perrucci MG, Del Gratta C, Romani GL, Corbetta M (2007) Electrophysiological signatures of resting state networks in the human brain. Proceedings of the National Academy of Sciences 104:13170–13175. 10.1073/pnas.0700668104 PMC1941820 17670949

[44] Kalcher K, Boubela RN, Huf W, Bartova L, Kronnerwetter C, et al**.** (2014) The spectral diversity of resting-state fluctuations in the human brain. PloS One 9. 10.1371/journal.pone.0093375 PMC3984093 24728207

[45] Bedo N, Ribary U, Ward LM (2014) Fast dynamics of cortical functional and effective connectivity during word reading. PloS One 9:e88940. 24551193 10.1371/journal.pone.0088940 PMC3925174

[46] Ito S, Rydygier P, Yeh FC, Hiolski E, Litke AM, et al**.** (2013) Co-existing frequency dependent functional networks in neuronal systems; San Diego, CA.

[47] Matsuda E, Mita T, Hubert J, Oka M, Bakkum D, et al**.** Multiple time scales observed in spontaneously evolved neurons on high-density CMOS electrode array; 2013.

[48] Ito S, Yeh FC, Hiolski E, Rydygier P, Gunning DE, et al (2014) Large-scale, high-resolution multielectrode-array recording depicts functional network differences of cortical and hippocampal cultures. PloS One 9:e105324. 25126851 10.1371/journal.pone.0105324 PMC4134292

[49] Schreiber T (2000) Measuring information transfer. Physical Review Letters 85:461–464. 10991308 10.1103/PhysRevLett.85.461

[50] Garofalo M, Nieus T, Massobrio P, Martinoia S (2009) Evaluation of performance of information theory-based methods and cross-correlation to estimate the functional connectivity in cortical networks. PloS One 4. 10.1371/journal.pone.0006482 PMC2715865 19652720

[51] Lizier JT, Heinzle J, Horstmann A, Haynes J, Prokopenko M (2011) Multivariate information-theoretic measures reveal directed information structure and task relevant changes in fMRI connectivity. Journal of Computational Neuroscience 30:85–107. 20799057 10.1007/s10827-010-0271-2

[52] Vicente R, Wibral M, Lindner M, Pipa G (2011) Transfer entropy - a model-free measure of effective connectivity for the neurosciences. Journal of Computational Neuroscience 30:45–67. 20706781 10.1007/s10827-010-0262-3 PMC3040354

[53] Stetter O, Battaglia D, Soriano J, Geisel T (2012) Model-free reconstruction of excitatory neuronal connectivity from calcium imaging signals. PLOS Computational Biology 8. 10.1371/journal.pcbi.1002653 PMC3426566 22927808

[54] Wibral M, Rahm B, Rieder M, Lindner M, Vicente R, et al (2011) Transfer entropy in magnetoencephalographic data: quantifying information flow in cortical and cerebellar networks. Progress in Biophysics and Molecular Biology 105:80–97. 21115029 10.1016/j.pbiomolbio.2010.11.006

[55] Ma C, Pan X, Wang R, Sakagami M (2013) Estimating causal interaction between prefrontal cortex and striatum by transfer entropy. Cognative Neurodynamics 7:253–261. 10.1007/s11571-012-9239-4 PMC3654150 24427205

[56] Gourevitch B, Eggermont JJ (2007) Evaluating information transfer between auditory cortical neurons. Journal of Neurophysiology 97:2533–2543. 17202243 10.1152/jn.01106.2006

[57] Shimono M, Beggs JM (2011) Spontaneous spike-trains reflect detailed topological properties of the structural neuronal network in vitro cortex.

[58] Shimono M, Beggs JM (2011) Mesoscopic neuronal activity and neuronal network architecture.

[59] Wollstadt P, Martinez-Zarzuela M, Vicente R, Diaz-Pernas FJ, Wibral M (2014) Efficient transfer entropy analysis of non-stationary neural time series. arXiv: 1401.4068v1. 10.1371/journal.pone.0102833 PMC4113280 25068489

[60] Zubler F, Gast H, Abela E, Rummel C, Hauf M, et al**.** (2014) Detecting functional hubs of ictogenic networks. Brain Topography 10.1007/s10548-014-0370-x. 10.1007/s10548-014-0370-x 24846350

[61] Besserve M, Scholkopf B, Logothetis NK, Panzeri S (2010) Causal relationships between frequency bands of extracellular signals in visual cortex revealed by an information theoretic analysis. Journal of Computational Neuroscience 29:547–566. 20396940 10.1007/s10827-010-0236-5 PMC2978901

[62] Shimono M, Beggs JM (2014) Functional clusters, hubs and communities in the cortical microconnectome. Cerebral Cortex. 10.1093/cercor/bhu252 PMC4585513 25336598

[63] Ito S, Hansen ME, Heiland R, Lumsdaine A, Litke AM, et al**.** (2011) Extending transfer entropy improves identification of effective connectivity in a spiking cortical network model. PloS One. pp. e27431. 10.1371/journal.pone.0027431 PMC3216957 22102894

[64] Lungarella M, Sporns O (2006) Mapping information flow in sensorimotor networks. PLOS Computational Biology. pp. e144. 10.1371/journal.pcbi.0020144 PMC1626158 17069456

[65] Eguiluz VE, Chialvo DR, Cecchi GA, Baliki M, Apkarian AV (2005) Scale-free brain functional networks. Physical Review Letters 94. 10.1103/PhysRevLett.94.018102 15698136

[66] Morgan RJ, Soltesz I (2008) Nonrandom connectivity of the epileptic dentate gyrus predicts a major role for neuronal hubs in seizures. Proceedings of the National Academy of Sciences 105:6179–6184. 10.1073/pnas.0801372105 PMC2299224 18375756

[67] Grinstein G, Linsker R (2005) Synchronous neural activity in scale-free network models versus random network models. Proceedings of the National Academy of Sciences 102:9948–9953. 10.1073/pnas.0504127102 PMC1175007 15998732

[68] Buzsaki G, Geisler C, Henze DA, Wang XJ (2004) Interneuron diversity series: Circuit complexity and axon wiring economy of cortical interneurons. Trends in Neuroscience 27:186–193. 10.1016/j.tins.2004.02.007 15046877

[69] Buckner RL, Sepulcre J, Talukdar T, Krienen FM, Liu H, et al (2009) Cortical hubs revealed by intrinsic functional connectivity: mapping, assessment of stability, and relation to Alzheimer’s disease. Journal of Neuroscience 29:1860–1873. 19211893 10.1523/JNEUROSCI.5062-08.2009 PMC2750039

[70] Eytan D, Marom S (2006) Dynamics and effective topology underlying synchronization in networks of cortical neurons. Journal of Neuroscience 26:8465–8476. 16914671 10.1523/JNEUROSCI.1627-06.2006 PMC6674346

[71] Kaiser M (2011) A tutorial in connectome analysis: topological and spatial features of brain networks. NeuroImage 57:892–907. 21605688 10.1016/j.neuroimage.2011.05.025

[72] Bonifazi P, Goldin M, Picardo MA, Jorquera I, Cattani A, et al**.** (2009) GABAergic hub neurons orchestrate synchrony in developing hippocampal networks. Science 326. 10.1126/science.1175509 19965761

[73] Timme N, Ito S, Myroshnychenko M, Yeh FC, Hiolski E, et al**.** Transfer entropy reveals time scale dependent networks in hippocampal and cortical cultures; 2013; Indianapolis, IN.

[74] Timme N, Ito S, Myroshnychenko M, Yeh FC, Hiolski E, et al**.** (2014) Multiplex networks of cortical and hippocampal neurons revealed at different timescales. BMC Neuroscience 15. 10.1371/journal.pone.0115764 PMC4275261 25536059

[75] Wibral M, Vicente R, Lizier JT, editors (2014) Directed information measures in neuroscience. Berlin: Springer-Verlag.

[76] van Wijk BCM, Stam CJ, Daffertshofer A (2010) Comparing brain networks of different size and connectivity density using graph theory. PloS One 5. 10.1371/journal.pone.0013701 PMC2965659 21060892

[77] van den Heuvel MP, Sporns O (2013) Network hubs in the human brain. Trends in Cognitive Sciences 17:683–696. 24231140 10.1016/j.tics.2013.09.012

[78] Rubinov M, Sporns O (2010) Complex network measures of brain connectivity: uses and interpretations. NeuroImage 52:1059–1069. 19819337 10.1016/j.neuroimage.2009.10.003

[79] Lee KM, Kim JY, Lee S, Goh KI (2014) Multiplex networks. In: D’Agostino G, Scala A, editors. Networks of networks: the last frontier of complexity. Switzerland: Springer International.

[80] Tomasi D, Volkow ND (2011) Functional connectivity hubs in the human brain. NeuroImage 57:908–917. 21609769 10.1016/j.neuroimage.2011.05.024 PMC3129362

[81] Crossley NA, Mechelli A, Scott J, Carletti F, Fox PT, et al (2014) The hubs of the human connectome are generally implicated in the anatomy of brain disorders. Brain 10.1093/brain/awu132:1–14. 10.1093/brain/awu132 PMC4107735 25057133

[82] Picardo MA, Guigue P, Bonifazi P, Batista-Brito R, Allene C, et al (2011) Pioneer GABA cells comprise a subpopulation of hub neurons in the developing hippocampus. Neuron 71:695–709. 21867885 10.1016/j.neuron.2011.06.018 PMC3163067

[83] Quilichini PP, Le Van Quyen M, Ivanov A, Turner DA, Carabalona A, et al (2012) Hub GABA neurons mediate gamma-frequency oscillations at ictal-like event onset in the immature hippocampus. Neuron 74:57–64. 22500630 10.1016/j.neuron.2012.01.026 PMC3328133

[84] Cossart R (2014) Operational hub cells: a morpho-physiologically diverse class of GABAergic neurons united by a common function. Current Opinion in Neurobiology 26:51–56. 24650504 10.1016/j.conb.2013.12.002

[85] Downes JH, Hammond MW, Xydas D, Spencer MC, Becerra VM, et al**.** (2012) Emergence of a Small-World Functional Network in Cultured Neurons. PLOS Computational Biology 8. 10.1371/journal.pcbi.1002522 PMC3355061 22615555

[86] Mountcastle VB (1997) The columnar organization of the neocortex. Brain 120:701–722. 9153131 10.1093/brain/120.4.701

[87] Meunier D, Lambiotte R, Bullmore ET (2010) Modular and hierarchically modular organization of brain networks. Frontiers in Neuroscience 4:1–11. 21151783 10.3389/fnins.2010.00200 PMC3000003

[88] Pouget A, Dayan P, Zemel R (2000) Information processing with population codes. Nature Reviews Neuroscience 1:125–132. 11252775 10.1038/35039062

[89] Johnson S, Torres JJ, Marro J, Munoz MA (2010) Entropic origin of disassortativity in complex networks. Physical Review Letters 104. 10.1103/PhysRevLett.104.108702 20366458

[90] Yuan WJ, Zhou C (2011) Interplay between structure and dynamics in adaptive complex networks: emergence and amplification of modularity by adaptive dynamics. Physical Review E 84. 10.1103/PhysRevE.84.016116 21867266

[91] Yuan WJ, Zhou JF, Li Q, Chen DB, Wang Z (2013) Spontaneous scale-free structure in adaptive networks with synchronously dynamical linking. Physical Review E 88. 10.1103/PhysRevE.88.022818 24032894

[92] Alivisatos AP, Chun M, Church GM, Greenspan RJ, Roukes ML, et al (2012) The Brain Activity Map Project and the Challenge of Functional Connectomics. Neuron 74:970–974. 22726828 10.1016/j.neuron.2012.06.006 PMC3597383

[93] Granger CWJ (1969) Investigating causal relations by econometric models and cross-spectral methods. econometrica 37:424–438.

[94] Geweke JF (1984) Measures of conditional linear dependence and feedback between time series. Journal of the American Statistical Association 79:907–915.

[95] Ding M, Chen Y, Bressler SL (2006) Granger causality: basic theory and application to neuroscience. In: Schelter B, Winterhalder M, Timmer J, editors. Handbook of time series analysis: recent theoretical developments and applications. Weinheim: Wiley-VCH. 437.

[96] Chavez M, Martinerie J, Le Van Quyen M (2003) Statistical assessment of nonlinear causality: application to epileptic EEG signals. Journal of Neuroscience Methods 124:113–128. 12706841 10.1016/s0165-0270(02)00367-9

[97] Saalmann YB, Pinsk MA, Wang L, Li X, Kastner S (2012) The pulvinar regulates information transmission between cortical areas based on attention demands. Science 337:753–756. 22879517 10.1126/science.1223082 PMC3714098

[98] Brovelli A, Ding M, Ledberg A, Chen Y, Nakamura R, et al (2004) Beta oscillations in a large-scale sensorimotor cortical network: directional influences revealed by Granger causality. Proceedings of the National Academy of Sciences 101:9849–9854. 10.1073/pnas.0308538101 PMC470781 15210971

[99] Nakhnikian A, Rebec GV, Grasse LM, Dwiel LL, Shimono M, et al**.** (2014) Behavior modulates effective connectivity between cortex and striatum. PloS One 9. 10.1371/journal.pone.0089443 PMC3949668 24618981

[100] Sharott A, Magill PJ, Bolam JP, Brown P (2005) Directional analysis of cohere oscillatory field potentials in the cerebral cortex and basal ganglia of the rat. Journal of Physiology 562:951–963. 15550466 10.1113/jphysiol.2004.073189 PMC1665537

[101] David O, Guillemain I, Saillet S, Reyt S, Deransart C, et al**.** (2008) Identifying neural drivers with functional MRI: an electrophysiological validation. PLOS Biology 6. 10.1371/journal.pbio.0060315 PMC2605917 19108604

[102] Amblard PO, Michel OJJ (2011) On directed information theory and Granger causality graphs. Journal of Computational Neuroscience 30:7–16. 20333542 10.1007/s10827-010-0231-x

[103] Friston KJ, Li B, Daunizeau J, Stephan KE (2011) Network discovery with DCM. NeuroImage 56:1202–1221. 21182971 10.1016/j.neuroimage.2010.12.039 PMC3094760

[104] Valdes-Sosa PA, Roebroeck A, Daunizeau J, Friston K (2011) Effective connectivity: influence, causality and biophysical modeling. NeuroImage 58:339–361. 21477655 10.1016/j.neuroimage.2011.03.058 PMC3167373

[105] Song C, Schwarzkopf DS, Lutti A, Li B, Kanai R, et al (2013) Effective connectivity within human primary visual cortex predicts interindividual diversity in illusory perception. Journal of Neuroscience 33:18781–18791. 24285885 10.1523/JNEUROSCI.4201-12.2013 PMC3841448

[106] Lorente V, Ferrandez-Vicente JM, Garrigos-Guerrero FJ, Lopez FdlP, Cuadra-Troncoso JM, et al**.** (2013) Neural spike activation in hippocampal cultures using Hebbian electrical stimulation. In: Ferrandez-Vicente JM, Alvarez-Sanchez JR, Lopez FdlP, Toledo-Moreo FJ, editors. IWINAC 2013. Mallorca, Spain.

[107] Tang A, Jackson D, Hobbs J, Chen W, Smith JL, et al (2008) A maximum entropy model applied to spatial and temporal correlations from cortical networks in vitro. Journal of Neuroscience 28:505–518. 18184793 10.1523/JNEUROSCI.3359-07.2008 PMC6670549

[108] Stoppini L, Buchs PA, Muller D (1991) A simple method for organotypic cultures of nervous tissue. Journal of Neuroscience Methods 37:173–182. 1715499 10.1016/0165-0270(91)90128-m

[109] Noraberg J, Poulsen FR, Blaabjerg M, Kristensen BW, Bonde C, et al (2005) Organotypic hippocampal slice cultures for studies of brain damage, neuroprotection and neurorepair. Current Drug Targets - CNS & Neurological Disorders 4:435–452. 16101559 10.2174/1568007054546108

[110] Pena F (2010) Organotypic cultures as tool to test long-term effects of chemicals on the nervous system. Current Medicinal Chemistry 17:987–1001. 20156165 10.2174/092986710790820679

[111] Zimmer J, Gahwiler BH (1984) Cellular and connective organization of slice cultures of the rat hippocampus and fascia dentata. Journal of Comparative Neurology 228:432–446. 6148364 10.1002/cne.902280310

[112] Gutierrez R, Heinemann U (1999) Synaptic reorganization in explanted cultures of rat hippocampus. Brain Research 815:304–316. 9878801 10.1016/s0006-8993(98)01101-9

[113] Muller D, Buchs PA, Stoppini L (1993) Time course of synaptic development in hippocampal organotypic cultures. Developmental Brain Research 71:93–100. 8432004 10.1016/0165-3806(93)90109-n

[114] Mielke JG, Comas T, Woulfe J, Monette R, Chakravarthy B, et al (2005) Cytoskeletal, synaptic, and nuclear protein changes associated with rat interface organotypic hippocampal slice culture development. Developmental Brain Research 160:275–286. 16271399 10.1016/j.devbrainres.2005.09.009

[115] Bausch SB, McNamara JO (2000) Synaptic connections from multiple subfields contribue to granule cell hyperexcitability in hippocampal slice cultures. Journal of Neurophysiology 84:2918–2932. 11110821 10.1152/jn.2000.84.6.2918

[116] Staal JA, Alexander SR, Liu Y, Dickson TD, Vickers JC (2011) Characterization of cortical neuronal and glial alterations during culture of organotypic whole brain slices from neonatal and mature mice. PloS One 6. 10.1371/journal.pone.0022040 PMC3137607 21789209

[117] Buchs PA, Stoppini L, Muller D (1993) Structural modifications associated with synaptic development in area CA1 of rat hippocampal organotypic cultures. Developmental Brain Research 71:81–91. 8432003 10.1016/0165-3806(93)90108-m

[118] Klostermann O, Wahle P (1999) Patterns of spontaneous activity and morphology of interneuron types in organotypic cortex and thalamus-cortex cultures. Neuroscience 92:1243–1259. 10426481 10.1016/s0306-4522(99)00009-3

[119] Gotz M, Bolz J (1992) Formation and preservation of cortical layers in slice cultures. Journal of Neurobiology 23:783–802. 1431845 10.1002/neu.480230702

[120] Bolz J, Novak N, Gotz M, Bonhoeffer T (1990) Formation of target-specific neuronal projections in organotypic slice cultures from rat visual cortex. Nature 346:359–362. 1695716 10.1038/346359a0

[121] Leiman AL, Seil FJ (1986) Influence of subcortical neurons on the functional development of cerebral neocortex in tissue culture. Brain Research 365:205–210. 3947990 10.1016/0006-8993(86)91631-8

[122] Baker RE, Pelt JV (1997) Cocultured, but not isolated, cortical explants display normal dendritic development: a long-term quantitative study. Developmental Brain Research 98:21–29. 9027401 10.1016/s0165-3806(96)00163-0

[123] Grewe BF, Helmchen F, Kampa BM (2014) Two-photon imaging of neuronal network dynamics in neocortex. In: Weber B, Helmchen F, editors. Optical imaging of neocortical dynamics: Humana Press. 133–150.

[124] Grewe BF, Langer D, Kasper H, Kampa BM, Helmchen F (2010) High-speed in vivo calcium imaging reveals neuronal network activity with near-millisecond precision. Nature Methods 7:339–349. 20400966 10.1038/nmeth.1453

[125] Sasaki T, Takahashi N, Matsuki N, Ikegaya Y (2008) Fast and accurate detection of action potentials from somatic calcium fluctuations. Journal of Neurophysiology 100:1668–1676. 18596182 10.1152/jn.00084.2008

[126] Royer S, Zemelman BV, Barbic M, Losonczy A, Buzsaki G, et al (2010) Multi-array silicon probes with integrated optical fibers: light-assisted perturbation and recording of local neural circuits in the behaving animal. European Journal of Neuroscience 31:2279–2291. 20529127 10.1111/j.1460-9568.2010.07250.x PMC2954764

[127] Deisseroth K (2011) Optogenetics. Nature Methods 8:26–29. 21191368 10.1038/nmeth.f.324 PMC6814250

[128] Litke AM, Bezayiff N, Chichilnisky EJ, Cunningham W, Dabrowski W, et al**.** (2004) What does the eye tell the brain?: development of a system for the large-scale recording of retinal output activity. IEEE Transactions on Nuclear Science 51.

[129] Wagenaar DA, Pine J, Potter SM (2006) An extremely rich repertoire of bursting patterns during the development of cortical cultures. BMC Neuroscience 7. 10.1186/1471-2202-7-11 PMC1420316 16464257

[130] Rolston JD, Wagenaar DA, Potter SM (2007) Precisely timed spatiotemporal patterns of neural activity in dissociated cortical cultures. Neuroscience 148:294–303. 17614210 10.1016/j.neuroscience.2007.05.025 PMC2096414

[131] Wibral M, Pampu N, Priesemann V, Siebenhuhner F, Seiwert H, et al**.** (2013) Measuring information-transfer delays. PloS One 8. 10.1371/journal.pone.0055809 PMC3585400 23468850

[132] Vakorin VA, Misic B, Krakovska O, McIntosh AR (2011) Empirical and theoretical aspects of generation and transfer of information in a neuromagnetic source network. Frontiers in Systems Neuroscience 5. 10.3389/fnsys.2011.00096 PMC3222882 22131968

[133] Kuhnert MT, Geier C, Elger CE, Lehnertz K (2012) Identifying important nodes in weighted functional brain networks: a comparison of different centrality approaches. Chaos 22. 10.1063/1.4729185 22757549

[134] Newman MEJ (2004) Fast algorithm for detecting community structure in networks. Physical Review E 69. 10.1103/PhysRevE.69.066133 15244693

[135] Leicht EA, Newman MEJ (2008) Community structure in directed networks. Physical Review Letters 100. 10.1103/PhysRevLett.100.118703 18517839

[136] Newman MEJ (2002) Assortative mixing in networks. Physical Review Letters 89. 10.1103/PhysRevLett.89.208701 12443515

[137] Benjamini Y, Yekutieli D (2001) The control of the false discovery rate in multiple testing under dependency. The Annals of Statistics 29:1165–1188.

[138] Benjamini Y, Hochberg Y (1995) Controlling the false discovery rate: a practical and powerful approach to multiple testing. Journal of the Royal Statistical Society B 57:289–300.

[139] Groppe DM, Urbach TP, Kutas M (2011) Mass univariate analysis of event-related brain potentials/fields I: A critical tutorial review. Psychophysiology 48:1711–1725. 21895683 10.1111/j.1469-8986.2011.01273.x PMC4060794

